# The KAT5-Acetyl-Histone4-Brd4 axis silences HIV-1 transcription and promotes viral latency

**DOI:** 10.1371/journal.ppat.1007012

**Published:** 2018-04-23

**Authors:** Zichong Li, Uri Mbonye, Zeming Feng, Xiaohui Wang, Xiang Gao, Jonathan Karn, Qiang Zhou

**Affiliations:** 1 Department of Molecular and Cell Biology, University of California, Berkeley, Berkeley, CA, United States of America; 2 Department of Molecular Biology and Microbiology, Case Western Reserve University, Cleveland, Ohio, United States of America; 3 School of Pharmaceutical Sciences, Xiamen University, Xiamen, Fujian, China; 4 Shenzhen Center for Disease Control and Prevention, Shenzhen, China; Duke University Medical Center, UNITED STATES

## Abstract

The bromodomain protein Brd4 promotes HIV-1 latency by competitively inhibiting P-TEFb-mediated transcription induced by the virus-encoded Tat protein. Brd4 is recruited to the HIV LTR by interactions with acetyl-histones3 (AcH3) and AcH4. However, the precise modification pattern that it reads and the writer for generating this pattern are unknown. By examining a pool of latently infected proviruses with diverse integration sites, we found that the LTR characteristically has low AcH3 but high AcH4 content. This unusual acetylation profile attracts Brd4 to suppress the interaction of Tat with the host super elongation complex (SEC) that is essential for productive HIV transcription and latency reversal. KAT5 (lysine acetyltransferase 5), but not its paralogs KAT7 and KAT8, is found to promote HIV latency through acetylating H4 on the provirus. Antagonizing KAT5 removes AcH4 and Brd4 from the LTR, enhances the SEC loading, and reverses as well as delays, the establishment of latency. The pro-latency effect of KAT5 is confirmed in a primary CD4+ T cell latency model as well as cells from ART-treated patients. Our data thus indicate the KAT5-AcH4-Brd4 axis as a key regulator of latency and a potential therapeutic target to reactivate latent HIV reservoirs for eradication.

## Author summary

A major impediment to the cure of HIV/AIDS is the viral latency. Previous studies have identified the bromodomain protein Brd4 as a promoter of HIV latency by binding to the viral LTR to inhibit Tat-induced transcription. Here, we discover that the LTR of latent HIV has low acetylated histone H3 (AcH3) but high AcH4 content, which recruits Brd4 to inhibit Tat-transactivation. Furthermore, the lysine acetyltransferase KAT5 but not the paralogs KAT7 and KAT8 promotes latency through acetylating H4 on the provirus. Antagonizing KAT5 removes AcH4 and Brd4 from the LTR, enhances loading of the Super Elongation Complex, and interferes with the establishment of latency. Thus, the KAT5-AcH4-Brd4 axis is a key regulator of HIV latency and a potential therapeutic target for eradicating latent HIV reservoirs.

## Introduction

The recent development of novel immunotherapeutic agents such as the bispecific dual-affinity retargeting (DART) antibodies that can engage a bound cytotoxic T cell to destroy an HIV-infected cell through recognizing the viral envelope proteins on the latter’s surface has fueled new optimism for finding a cure for HIV/AIDS[1]. However, a major impediment to the cure effort is the latent viral reservoirs in long-lived CD4+ T cells that do not express any HIV protein/RNA, and thus cannot be recognized either by DART or by the host immune system[2].

HIV latency is the result of silenced proviral transcription due to multiple complementary mechanisms[3]. To expose the latent reservoirs for accelerated clearance, numerous “latency-reversing agents” (LRAs) have been identified that target specific stages of the HIV transcription cycle[4]. However, extensive *ex vivo* studies suggest that individual LRAs are not very potent and that combinations of LRAs will be required to effectively purge the latent reservoirs[5]. Among the successful combinatorial *ex vivo* trials, inhibition of the BET bromodomain protein Brd4 with JQ1 strongly synergized with other LRAs to reverse viral latency[6,7]. Although these *ex vivo* studies provide an important proof of concept, the available LRAs are either highly toxic or yet to be proven efficacious in clinical settings[3]. Thus, better and safer LRAs are urgently needed.

Brd4 is known to use its two bromodomains to bind to acetylated histones H3 and H4 (AcH3 and AcH4)[8] and its C-terminal PID (P-TEFb-interacting domain) to recruit the human positive transcription elongation factor b (P-TEFb) to chromatin[9,10] to promote transcription of cellular primary response genes[11]. Counterintuitively, during activation of HIV transcription by the viral-encoded Tat protein, Brd4 acts as a potent inhibitor[10,12]. This is because Brd4, which is highly abundant *in vivo*, can directly compete with Tat for binding to the limited cellular supply of P-TEFb[10,12], the protein kinase that is an integral component of the multi-subunit Super Elongation Complex (SEC) used by Tat as a host cofactor for HIV transactivation[13,14]. By removing Brd4 from the HIV long terminal repeat (LTR), JQ1 and other bromodomain inhibitors have been shown to reverse HIV latency by promoting the Tat-SEC formation, and consequently, Tat-transactivation[15,16]. Antagonizing the Tat-SEC complex may not be the only way that Brd4 controls HIV latency. A recent study has shown that the short isoform of Brd4 (Brd4S) that lacks the PID can instead recruit the repressive SWI/SNF chromatin-remodeling complexes onto the latent HIV-1 promoter to repress transcription[17].

Given that targeting Brd4 can be a very effective strategy to purge latent HIV reservoirs[6,7], it is important to identify the prevailing histone acetylation pattern that allows Brd4 to be recruited and persistent on the HIV provirus. Knowing the exact Brd4 target on HIV chromatin and how it is created during latency establishment may help us devise more specific and efficient methods to displace Brd4 from the LTR for latency reversal. This will also help resolve the paradox that histone acetylation, which is typically associated with relaxed chromatin structure and HIV transactivation[18], can be used to attract Brd4 to silence proviral transcription. We therefore examined the status of AcH3 and AcH4 present on the LTR of a pool of latent HIV proviruses that had diverse integration sites. Our data indicate that upon entering latency, these proviruses displayed elevated levels of AcH4 and Brd4 but drastically reduced levels of AcH3 on their LTR, raising the possibility that AcH4 is the primary histone modification responsible for attracting and retaining Brd4 on the silenced proviruses.

Three major histone acetyltransferases (HATs) are known to modify H4: KAT5 (Tip60) is known to acetylate lysines 5, 8, 12 and 16[19]; KAT7 targets three out of the four positions; and KAT8 (MOF) modifies lysine16 exclusively[20]. Our data indicate that downregulating the expression or activity of KAT5, but not KAT7 and KAT8, removed AcH4 and Brd4 from the HIV LTR, leading to the enhanced SEC loading, Tat-transactivation and escape from latency. Consistent with these results, our data further show that silencing KAT5 interfered with the establishment of latency. Together, these results have demonstrated a critical role for KAT5 and its acetylation of H4 in promoting Brd4’s recruitment to the HIV LTR and establishment of latency. Furthermore, they reveal that inhibiting the KAT5-AcH4-Brd4 axis is potentially an effective strategy for activating the latent proviruses for subsequent eradication.

## Results

### Silenced HIV proviruses contain elevated amounts of AcH4 and Brd4 but drastically decreased levels of AcH3

Given the importance of Brd4 in modulating HIV latency, we determined the precise histone acetylation pattern on the viral LTR, which is targeted by Brd4 to sequester P-TEFb away from the Tat-SEC complex. Toward this goal, we first examined the levels of AcH3 and AcH4 on the LTR of latent HIV proviruses.

**Fig 1:**
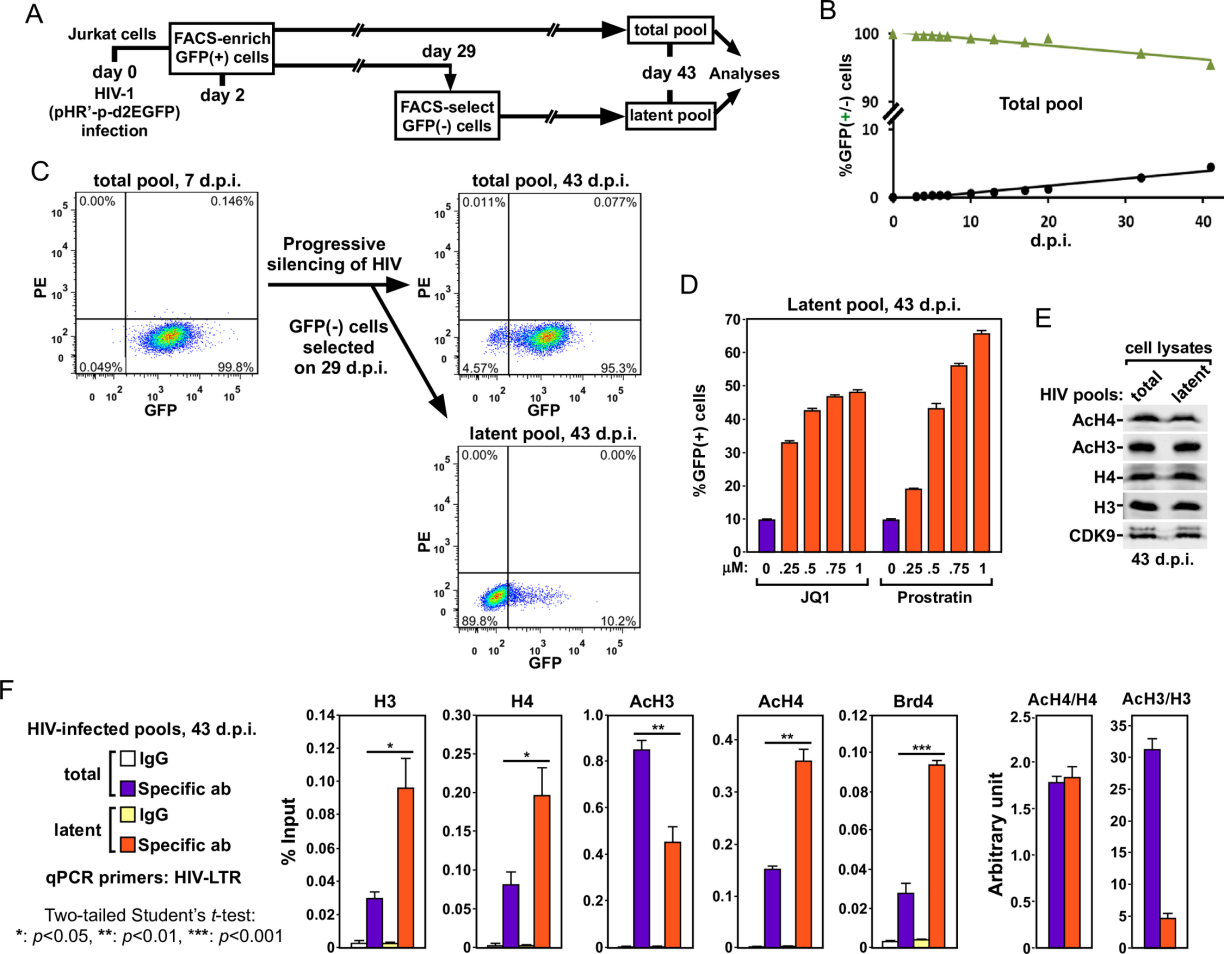
Establishment of HIV latency correlates with elevated amounts of AcH4 and Brd4 but drastically decreased AcH3 content on viral LTR. **A.** A schematic diagram showing the procedure and timeline of experiments to progressively establish HIV latency in the total infected Jurkat cell pool (total pool) as well as to enrich the latently infected cell pool (latent pool). **B.** The total pool was checked by fluorescence-activated cell sorting (FACS) for the percentages of GFP(+) or (-) cells on the indicated days post infection (d.p.i.) and the average of three measurements at each time point is shown. **C.** Representative FACS plots of total and latent pools at the indicated time points. **D.** The latent pool was treated in triplicates on 43 d.p.i. by JQ1 or prostratin at the indicated concentrations for 24 hr and subjected to FACS to determine the percentage of GFP(+) cells in each cell population. The error bars indicate mean +/- SD from three independent measurements. **E.** Western analysis of the total and latent pools on 43 d.p.i. for the various proteins labeled on the left. **F.** The total and latent pools on 43 d.p.i. were subjected to ChIP-qPCR analyses to determine the levels of the indicated proteins bound to HIV LTR. The ChIP signals were normalized to those of input DNA and shown as an average of three independent reactions, with the error bars representing mean +/- SD and the asterisks indicating the levels of statistical significance calculated by two-tailed Student’s *t*-test.

The original observations indicating the importance of Brd4 in HIV latency were made by using isolated clones of Jurkat T cells (e.g. the J-Lat cell lines and the 2D10 system[12,15,16]) that contain only a single viral integration site within each cell population. To rule out site-specific integration effects, we created a pool of latently infected cells that contain a diverse array of all possible integration sites. This was achieved by adapting a protocol from Pearson *et al*. [21] to progressively establish HIV latency in Jurkat cells that were first infected on day 0 with a GFP-encoding HIV virus (Fig 1A). The freshly infected cells containing actively replicating HIV were then sorted by fluorescence-activated cell sorting (FACS) on day 2 into a GFP(+) population, which was then cultured for additional 6 weeks (Fig 1A). This population of cells, called the total pool, was examined for the percentages of GFP(+) cells on various days post infection (d.p.i.). The results showed that on 43 d.p.i., 95.3% of the cells still remained GFP(+), while the rest have reverted to the GFP(-) state (Fig 1B and 1C).

To enrich the latently infected cells, a portion of the GFP(+) cells that was originally isolated by FACS on 2 d.p.i. was subjected to sorting again on 29 d.p.i. to isolate the GFP(-) cells, which were then allowed to propagate until 43 d.p.i. (Fig 1A). This population, called the latent pool, had 89.8% of cells that remained GFP(-) on 43 d.p.i., while the rest had undergone spontaneous reactivation to become GFP(+) again (Fig 1C). It is important to point out that the HIV proviruses in the latent pool could readily be reactivated by conventional LRAs such as JQ1 and prostratin, demonstrating that the pool retained transcriptionally functional proviruses (Fig 1D).

Western analysis of cell lysates indicates no major difference in the overall levels of AcH3, AcH4, total H3 and total H4 between the total and latent pools (Fig 1E). However, examination by chromatin immunoprecipitation (ChIP) demonstrates a 2 to 3-fold increase in the levels of total H3 and H4 on the HIV LTR in the latent pool compared to the total pool (Fig 1F). This is consistent with the previous finding that the latent, transcriptionally silent HIV proviruses are more likely to be occupied by nucleosome 1 (nuc-1) situated immediately downstream of the transcription start site[22]. Despite the increased total H3 level on the viral LTR in the latent pool, the acetylation of H3 decreased by 2-fold (Fig 1F). This observation agrees with the general view that deacetylated H3 provides a marker for repressed and compact chromatin structure[23] as well as the result obtained previously using the Jurkat 2D10 cells, a widely used post-integrative HIV latency model[21].

In contrast to AcH3, the AcH4 level on the LTR increased when the pool of proviruses entered latency (Fig 1F). In fact, AcH4 increased at about the same extent as total H4 during this process. Similarly, more Brd4 was found on the LTR in the latent pool than in the total pool (Fig 1F), likely due to the increased AcH4 level. Collectively, these data indicate that during the establishment of HIV latency, the incoming nucleosomes assembled on the viral LTR had very low AcH3 content, but maintained a high AcH4 level, which in turn increased the recruitment of Brd4 to inhibit HIV gene expression.

### Antagonizing KAT5 reverses HIV latency and potentiates conventional LRAs

Previously, it has been reported that Brd4 recognizes both AcH3 and AcH4 to interact with a chromatin template[8]. However, the surprising finding that AcH4, but not AcH3, exists in high concentration on the silenced HIV LTR raises the possibility that AcH4 is the primary histone modification responsible for recruiting Brd4 onto the LTR.

Lysines 5, 8, 12 and 16 (H4K5/8/12/16) are the primary acetylation sites found at the N-terminus of H4 [24] and acetylation of these sites has been shown to promote the binding of Brd4 to H4 both *in vivo* and *in vitro* [8]. Three major histone acetyltransferases (HATs) are known to modify H4. While KAT5 (Tip60) is the only one capable of acetylating all four H4K5/8/12/16 positions, the other KATs are more selective [20,24]. For example, KAT7 acetylates H4K5, 8 and 12, whereas KAT8 only acetylates H4K16. We therefore examined the impact of silencing the expression of KAT5, KAT7 or KAT8 on HIV transcription and latency.

**Fig 2:**
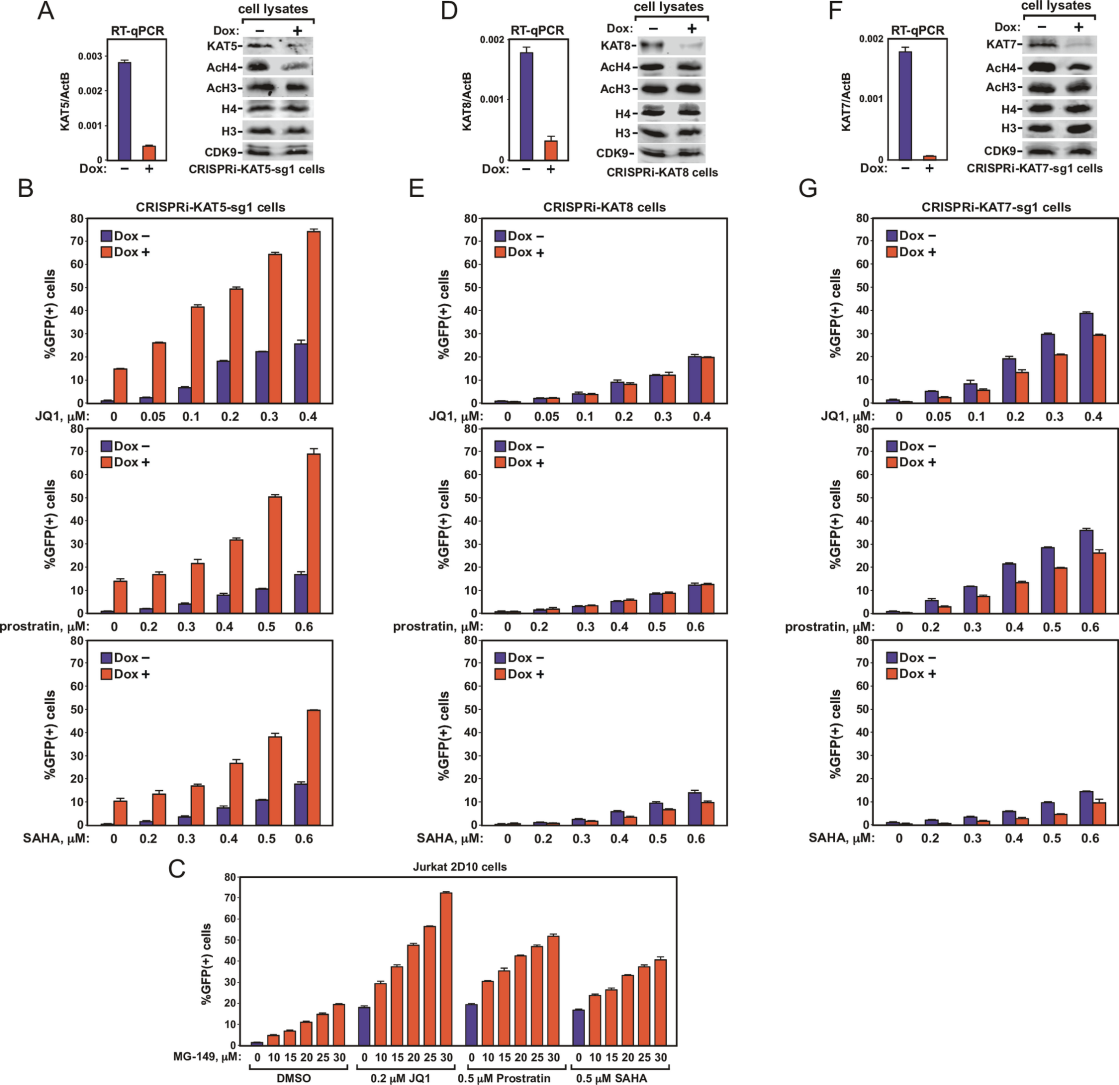
Antagonizing KAT5 but not KAT7 or KAT8 reverses HIV latency and potentiates conventional LRAs. **A. D.** & **F.** The Jurkat 2D10-based inducible CRISPRi-KAT5-sg1, CRISPRi-KAT7-sg1, and CRISPRi-KAT8 cells were treated with (+) or without (-) doxycycline (Dox) and analyzed by RT-qPCR for the KAT5/7/8 mRNA levels, which were normalized to those of ActB, and by Western blotting for the indicated proteins. **B. E.** & **G.** CRISPRi-KAT5-sg1, CRISPRi-KAT7-sg1, and CRISPRi-KAT8 cells were treated with or without Dox (1 μl/ml) and the various LRAs at the indicated concentrations. The treated cells were subjected to FACS analysis to determine the percentage of GFP(+) cells in each cell population. **C.** Wild-type 2D10 cells were treated with combinations of MG-149 and DMSO or the various LRAs at the indicated concentrations and subjected to FACS analysis as in B. Each column in all panels represents the average of three independent measurements, with the error bars indicating mean +/- SD.

We used the doxycycline (Dox)-inducible CRISPRi system[25] to suppress the expression of KAT5, KAT7 or KAT8 in the Jurkat-based 2D10 cell line, a widely used post-integrative HIV latency model containing the GFP-coding sequence in place of the viral *Nef* gene[21]. An sgRNA sequence (sg1) that specifically targets the promoter region of the KAT5 gene was found to reduce the KAT5 mRNA level by ~80% in the engineered CRISPRi-KAT5-sg1 cells upon exposure to Dox (Fig 2A, left panel). Western analysis of the cell lysates showed a corresponding decrease in the KAT5 protein level as well as a marked reduction of the AcH4 but not AcH3 level (Fig 2A, right panel). The global AcH4 reduction agrees well with the demonstrated role of KAT5 as a promiscuous acetyltransferase for H4[19].

Using GFP induction as an indicator of latency reversal in the engineered 2D10 cells, the FACS analyses show that the Dox-induced inhibition of KAT5 expression by CRISPRi caused ~15% of the cell population to become GFP(+) (Fig 2B). In addition, CRISPRi against KAT5 also strongly synergized with JQ1, prostratin, and SAHA, the three well-studied conventional LRAs, to enhance GFP production across a broad range of drug concentrations (Fig 2B). Very similar results were obtained in another 2D10-based CRISPRi-KAT5 cell pool, in which the inhibition of KAT5 expression was achieved by using sgRNA #2 (sg2) that targets a distinct KAT5 promoter sequence (S1 Fig), thus effectively ruling out a potential off-target effect caused by CRISPRi. Finally, inhibition of the catalytic activity of KAT5 with a selective inhibitor MG-149[26] also reversed HIV latency and potentiated traditional LRAs in a dosage-dependent manner (Fig 2C).

### CRISPRi inhibition of KAT7 or KAT8 failed to reactivate latent HIV

In contrast to the above results showing a key role of KAT5 in silencing HIV transcription, the CRISPRi-mediated inhibition of the KAT7 or KAT8 expression in 2D10 cells (Fig 2D–2G) neither activated GFP expression by itself nor promoted the effects of the three conventional LRAs (Fig 2E and 2G). In fact, the inhibition of KAT7 even slightly decreased the levels of both basal and the LRA-induced GFP production (Fig 2G), a result that was also observed in another CRISPRi-KAT7 cell pool generated with a different sgRNA (S2 Fig). Thus, unlike KAT5, KAT7 appears to play a small but positive role in HIV transcription.

Consistent with the demonstration of KAT8 as a specific H4K16 acetyltransferase and KAT7 as a HAT for three out of the four lysine positions in the H4 N-terminus[20,24], the global AcH4 level was only slightly decreased in the CRISPRi-KAT8 cells but more prominently decreased in the CRISPRi-KAT7-sg1 cells (Fig 2D & 2F). Collectively, these data indicate that KAT5, but not its two paralogs KAT7 and KAT8, is required to maintain the HIV provirus in a transcriptionally silent state in latency.

### Antagonizing KAT5 activates Tat-dependent HIV transcriptional elongation but inhibits cellular primary response genes

**Fig 3:**
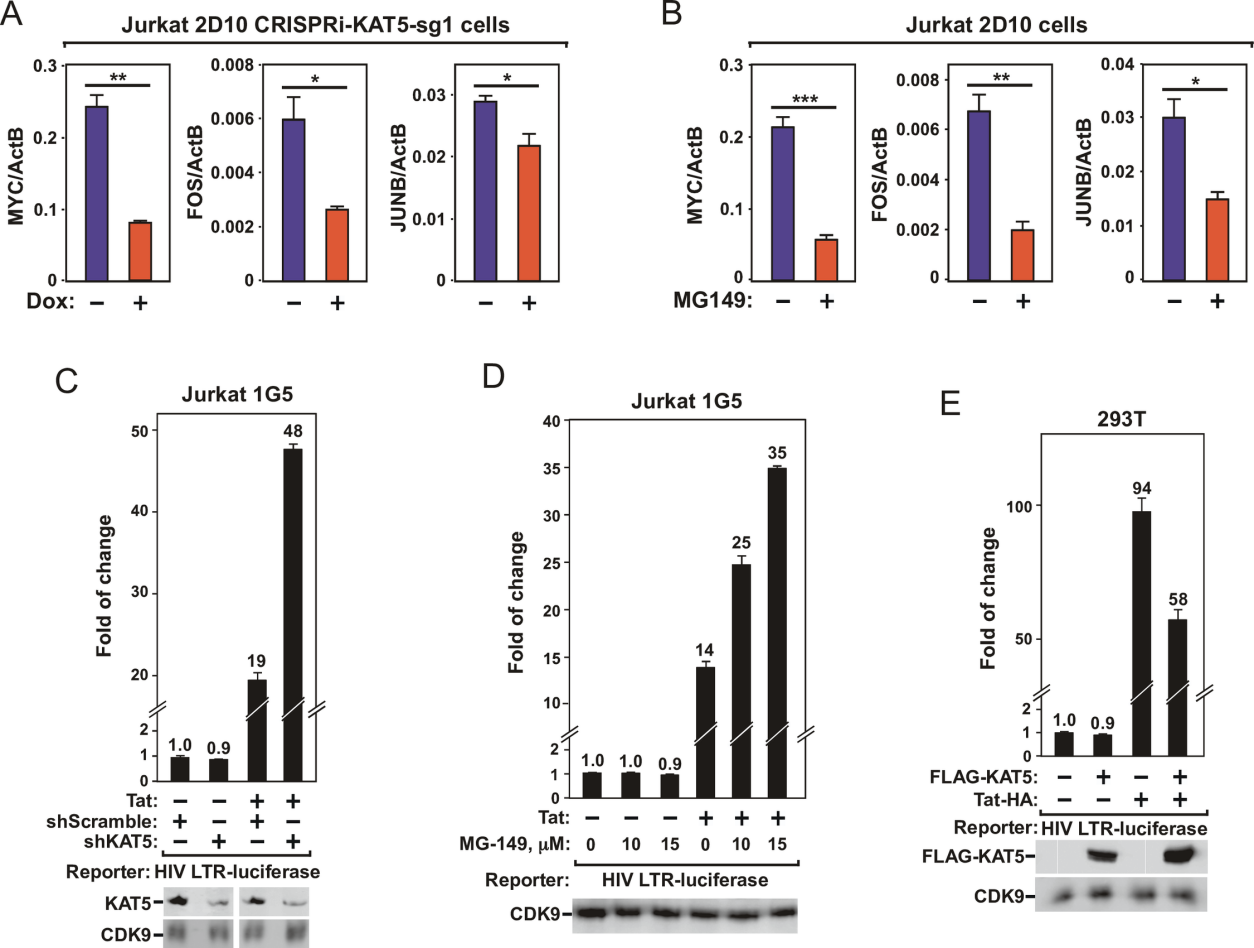
Antagonizing KAT5 activates Tat-dependent HIV transcription but inhibits cellular primary response genes. **A.** & **B.** Jurkat 2D10-based CRISPRi-KAT5-sg1 (A) and parental 2D10 (B) cells were treated with (+) or without (-) Dox (A) or MG-149 (B). The mRNA levels of the indicated genes were measured by RT-qPCR and normalized to those of ActB and shown as averages of three independent measurements. The error bars indicate mean +/- SD and the asterisks denote levels of statistical significance calculated by two-tailed Student’s *t*-test (*: p<0.05, **: p<0.01, and ***: p<0.001). **C.** & **D.** Luciferase activities were measured in extracts of Jurkat 1G5 or Jurkat 1G5+Tat cells containing an integrated HIV-1 LTR-luciferase reporter construct and stably expressing the indicated shRNA (C) or treated with the indicated concentrations of MG-149 (D). **E.** Luciferase activities were measured in extracts of 293T cells that were transfected with the HIV-1 LTR-luciferase reporter construct together with the vector expressing FLAG-KAT5- and/or Tat-HA as indicated. All experiments in C to E were performed in triplicates with the error bars representing mean +/- SD and the activities in the first column set to 1.0. An aliquot of each cell extract was examined by Western blotting for the proteins labeled on the left.

In the majority of cases, acetyltransferases activate gene expression through acetylating histone tails, which de-condenses chromatin to facilitate transcriptional initiation and elongation[23]. The Brd4-P-TEFb complex that is recruited to a chromatin template through the interaction between the Brd4 bromodomains and acetyl-histones plays a critical role in promoting transcriptional elongation of many cellular genes, especially those that are involved in primary responses[27,28]. In light of our surprising finding that KAT5 did not activate HIV, but rather repressed HIV gene expression to keep the virus in latency, we investigated how antagonizing KAT5 might affect the expression of non-HIV genes such as MYC, FOS and JUNB, which are well-known cellular primary response genes[11,29]. In agreement with the expectation that the pan acetyltransferase KAT5 should act to promote gene expression, inhibiting KAT5’s expression by CRISPRi (Fig 3A) or activity by MG-149 (Fig 3B) was found to decrease the mRNA levels of all the three genes.

To confirm that the inhibition of HIV gene expression by KAT5 was indeed working through the viral LTR and at the transcription level, we first examined the effect of the shRNA-induced KAT5 knockdown (KD) on the ability of an integrated HIV-1 LTR to drive the expression of the luciferase reporter gene in Jurkat 1G5 cells[30]. Interestingly, while the KD slightly decreased the basal LTR activity in the absence of Tat, it significantly increased luciferase expression when Tat was present in the cells (Fig 3C). The differential effect on basal versus Tat-dependent HIV LTR activity was also observed when the catalytic activity of KAT5 was inhibited by MG-149 (Fig 3D). In contrast to the effects caused by antagonizing KAT5, the overexpression of KAT5 significantly repressed the Tat-dependent, but not -independent LTR activity (Fig 3E). Finally, using a qRT-PCR-based assay that can distinguish between the processes of transcription initiation and elongation[31], the inhibitory effect of KAT5 on Tat-transactivation was deemed to be primarily at the elongation stage (S3 Fig). All together, these data strongly suggest that while KAT5 plays a stimulatory role in promoting the expression of cellular Brd4-dependent primary response genes, it can efficiently inhibit the Tat-dependent HIV transcriptional elongation.

### Antagonizing KAT5 reduces AcH4, but not AcH3, on both HIV and cellular promoters

**Fig 4:**
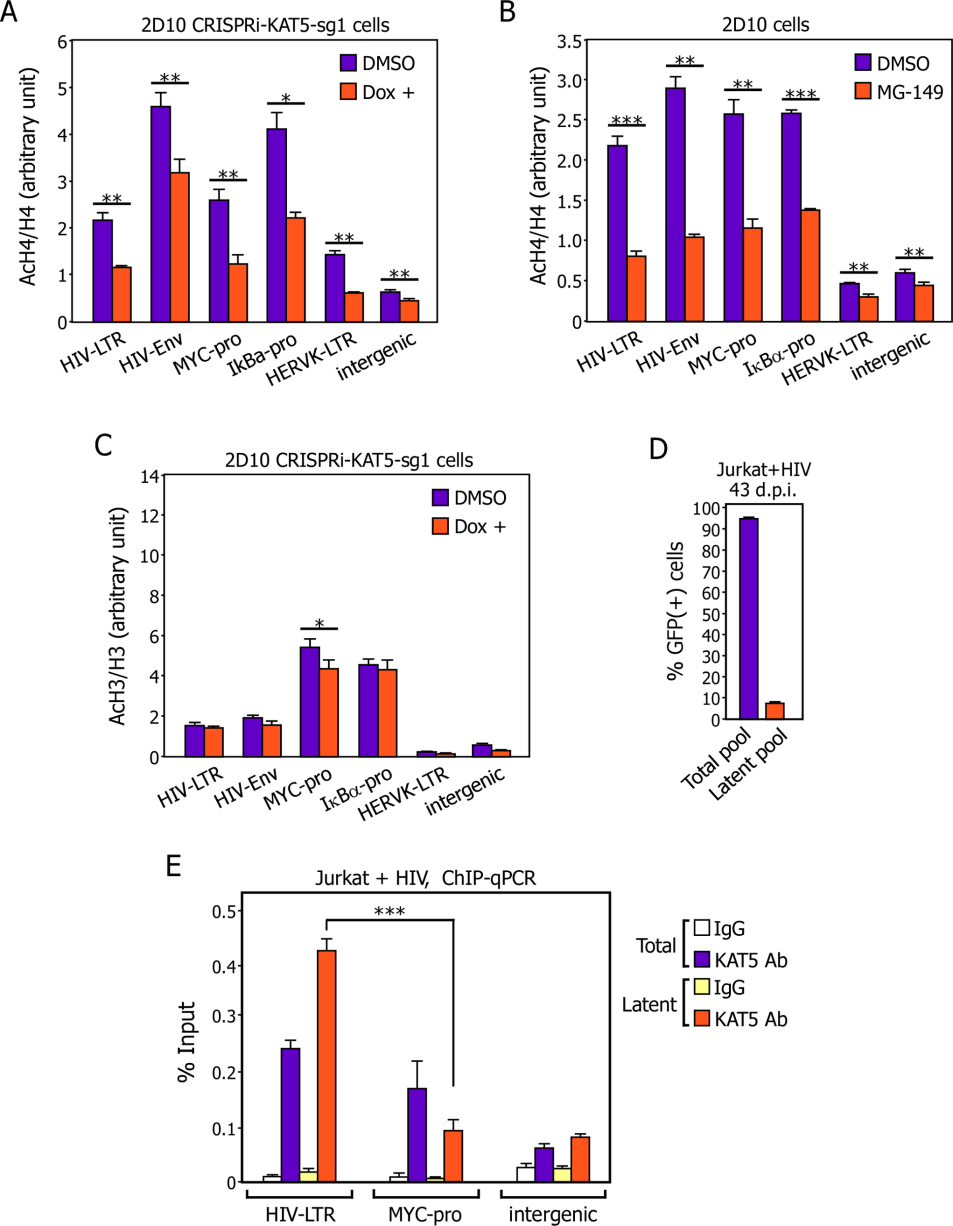
Antagonizing KAT5 reduces AcH4 but not AcH3 level on both HIV and non-HIV gene promoters and a higher level of KAT5 exists on the viral LTR than on the cellular MYC gene promoter in latently infected cells. **A., B., & C.** The 2D10-based CRISPRi-KAT5-sg1 (A & C) and parental 2D10 (B) cells were treated with the indicated drugs and subjected to ChIP-qPCR assays to determine the levels of AcH3, AcH4, total H3 and total H4 bound to the various genomic locations labeled at the bottom. The ChIP-qPCR signals were normalized to those of input DNA for each genomic location and the ratios of AcH3 over H3 and AcH4 over H4 were shown. The error bars represent mean +/- SD from three independent qPCR reactions. The asterisks (*: p<0.05, **: p<0.01, and ***: p<0.001) indicate different levels of statistical significance as calculated by two-tailed Student’s t-tests. **D.** The total and latently infected Jurkat cell pools generated in Fig 1A were analyzed by FACS to determine the percentage of GFP(+) cells in each population. The error bars indicate mean +/- SD from three independent measurements. **E.** The indicated cell pools from D were subjected to ChIP-qPCR analysis with the anti-KAT5 antibody to determine the levels of KAT5 bound to the various genomic locations labeled at the bottom. The ChIP-qPCR signals were normalized to those of input DNA for each location and shown as an average of three independent reactions, with the error bars representing mean +/- SD and the asterisks indicating the levels of statistical significance calculated by two-tailed Student’s *t*-test.

To determine whether the global reduction of the AcH4 but not AcH3 level observed in the CRISPRi-KAT5-sg1 cell lysates (Fig 2A) would also result in a decreased AcH4 but not AcH3 level on both HIV and cellular gene promoters, we conducted the anti-AcH4 and -AcH3 ChIP assay. Indeed, suppressing KAT5’s expression by CRISPRi (Fig 4A) or activity by MG-149 (Fig 4B) reduced the level of AcH4 detected on the HIV provirus at both the viral LTR and *Env* gene. In addition, a marked reduction in the AcH4 level was also detected at the promoters of two cellular genes, MYC and IκBα, as well as at the endogenous retroviral element HERVK[32] and an intergenic region upon the inhibition of KAT5 (Fig 4A and 4B).

In contrast, CRISPRi against KAT5 did not significantly affect the AcH3 levels at these HIV and non-HIV locations, with the only exception seen at the MYC promoter, where a small reduction that could be a secondary effect of the diminished transcription was detected (Fig 4C). Furthermore, on the silent HIV provirus in 2D10 cells, the overall levels of AcH3 relative to total H3 were lower than those detected on the two cellular genes MYC and IκBα (Fig 4C), a result that echoes the observation made in a whole population of latently infected Jurkat cells in Fig 1F. Finally, the poorly transcribed HERVK and the intergenic genomic region displayed generally low levels of AcH3 and AcH4 when compared with the robustly expressed HIV, MYC and IκBα genes (Fig 4C & 4B).

While the relatively low level of AcH3 on the latent HIV LTR has been reported previously [21,33] and is likely due to a diminished recruitment of H3 acetyltransferase p300 [34], the unusually high level of AcH4 on the LTR (Figs 1F, 4A and 4B) is yet to be explained. To this end, we compared the levels of KAT5 on the HIV LTR and the MYC promoter in infected cells. The ChIP data demonstrate that the LTR had a mildly higher level of KAT5 than did the MYC promoter in the total pool of infected cells. However, the difference between the two became more pronounced and statistically significant upon the establishment of viral latency (Fig 4D and 4E). Thus, the unbalanced loadings of p300 and KAT5 likely contribute to the unusually low AcH3 but high AcH4 level on the latent HIV LTR.

### Inhibition of KAT5 selectively removes Brd4 from and increases SEC binding to the HIV provirus

Since inhibition of KAT5 led to an overall reduction of the AcH4 level at both the HIV and non-HIV gene promoters, we asked how the reduced AcH4 level could promote Tat-dependent HIV transcription while at the same time suppress the expression of cellular primary response genes. To answer this question, we investigated the consequence of inhibiting KAT5 on the binding of Brd4 and the human Super Elongation Complex (SEC) to the HIV and non-HIV loci.

**Fig 5:**
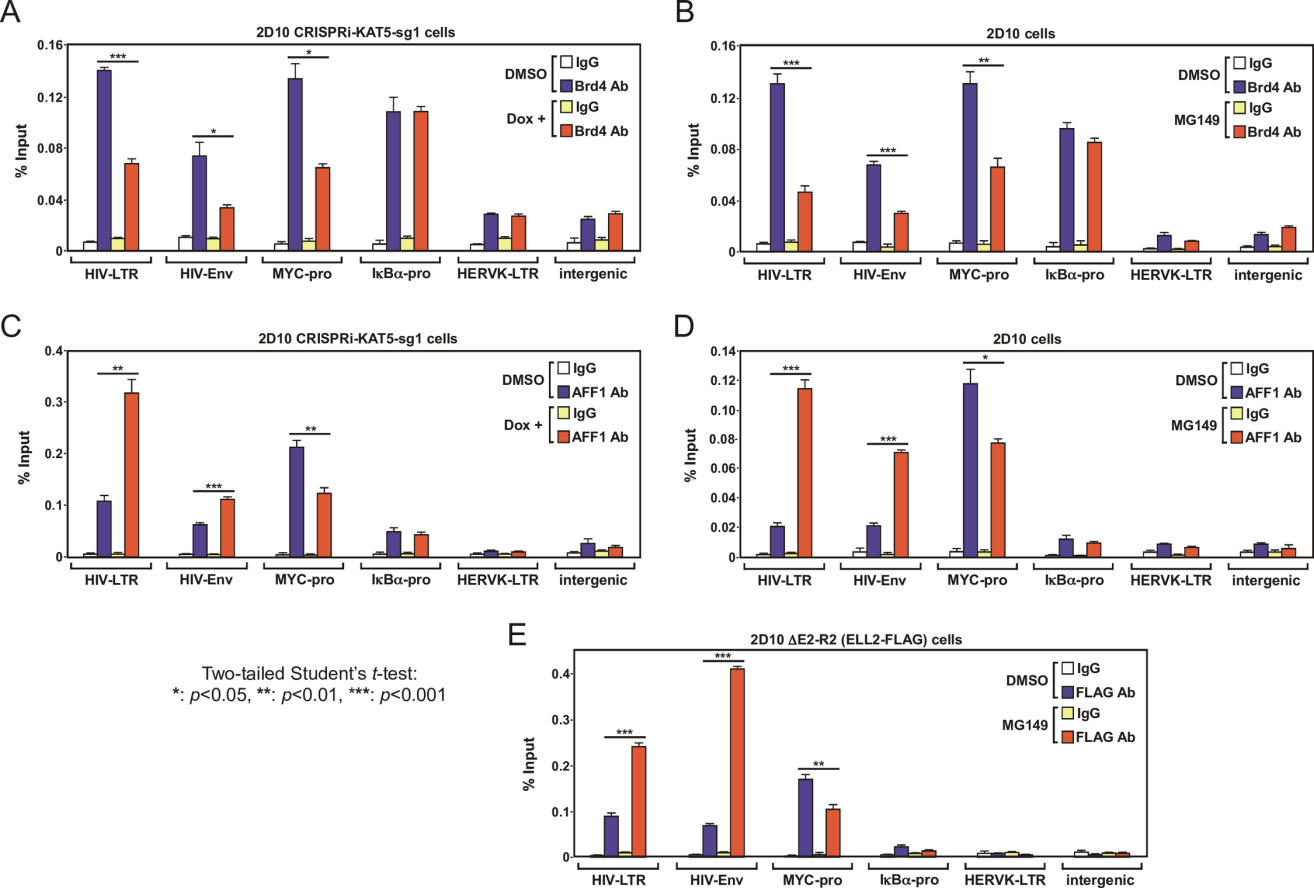
Inhibition of KAT5 selectively removes Brd4 from and increases SEC binding to the HIV provirus. **A., B., C., D., & E.** CRISPRi-KAT5-sg1 (A & C), 2D10 (B & D), and ΔE2-R2 (E) cells were treated with DMSO or the indicated drugs and subjected to ChIP-qPCR assays using the antibodies (Ab) that specifically recognize the indicated proteins bound to the various genomic regions as labeled at the bottom. The ChIP-qPCR signals were normalized to those of input DNA for each genomic location. The error bars represent mean +/- SD from three independent qPCR reactions. The asterisks indicate different levels of statistical significance as calculated by two-tailed Student’s t-tests.

The ChIP assay conducted in the inducible CRISPRi-KAT5-sg1 cells indicates that upon the Dox-induced down-regulation of KAT5, the Brd4 level significantly decreased at the HIV LTR, HIV *Env*, and the MYC promoter region (Fig 5A). This implicates AcH4 as the primary route of recruitment for Brd4 at these locations. In contrast, although the Brd4 level was quite high at the IκBα gene promoter before the Dox treatment, it displayed little change after CRISPRi silencing of KAT5 (Fig 5A), probably because Brd4 is recruited to this locus by recognizing mostly AcH3 but not AcH4. The Brd4 levels at the poorly transcribed HERVK and the intergenic region were relatively low and remained fairly constant upon CRISPRi silencing of KAT5 (Fig 5A). Importantly, a very similar change in the Brd4 distribution pattern was also observed when the catalytic activity of KAT5 was inhibited by MG-149 (Fig 5B).

A recent study by the Ott laboratory demonstrates that both the long and short isoforms of Brd4 can promote HIV latency, albeit through distinct mechanisms [17]. To assess the roles of the two Brd4 isoform in transducing the signal downstream of the KAT5-AcH4 axis, we compared the effect of MG-149 on the bindings of the long (simply labeled as Brd4 throughout the current study) and short form of Brd4 (Brd4S) to the integrated HIV-1 LTR. The ChIP data show that when expressed at a similar level, more Brd4 bound to the LTR than did Brd4S (S4 Fig). Furthermore, compared to Brd4S, the binding of Brd4 to the LTR was also more sensitive to the MG-149-induced AcH4 reduction. Thus, between the two Brd4 isoforms, the long form appears to be more responsive to changes in the level of AcH4 on the LTR and thus more likely to be a key target of the KAT5-AcH4 axis.

Because the abundant Brd4 long isoform containing the C-terminal P-TEFb-interacting domain is a direct competitor of HIV Tat for the limited cellular supply of P-TEFb[10,12], the decreased Brd4 occupancy on the HIV provirus in KAT5-inhibited cells is expected to free up P-TEFb for its incorporation into the Tat-SEC complex, which is very efficiently recruited to the provirus through the TAR RNA route [16,35]. Consistent with this mechanism, the ChIP analysis showed that inhibiting KAT5’s expression by CRISPRi (Fig 5C) or activity by MG-149 (Fig 5D) significantly increased the level of AFF1, the scaffolding subunit of the SEC, on both the HIV LTR and *Env* gene. In addition, MG-149 also significantly enhanced the loading of another key SEC subunit ELL2 onto the HIV provirus in an engineered Jurkat cell line ΔE2-R2 (Fig 5E), in which the endogenous ELL2 gene has been knocked out and ELL2-FLAG is expressed at a similar level from an integrated vector[31].

By contrast, the inhibition of KAT5 decreased the SEC level on the MYC promoter (Fig 5C–5E), which agrees with the diminished MYC expression in the KAT5-inhibited cells (Fig 3A & 3B). Although the precise function of SEC in MYC transcription is yet to be revealed, our data suggest a likely role for Brd4 in recruiting SEC to this important primary response gene. Finally, very low levels of AFF1 and ELL2-FLAG were detected at the IκBα gene promoter, HERVK, and the intergenic region (Fig 5C–5E), implicating that the SEC plays little role in their transcription.

### KAT5 depletion prevents HIV from efficiently establishing latency

**Fig 6:**
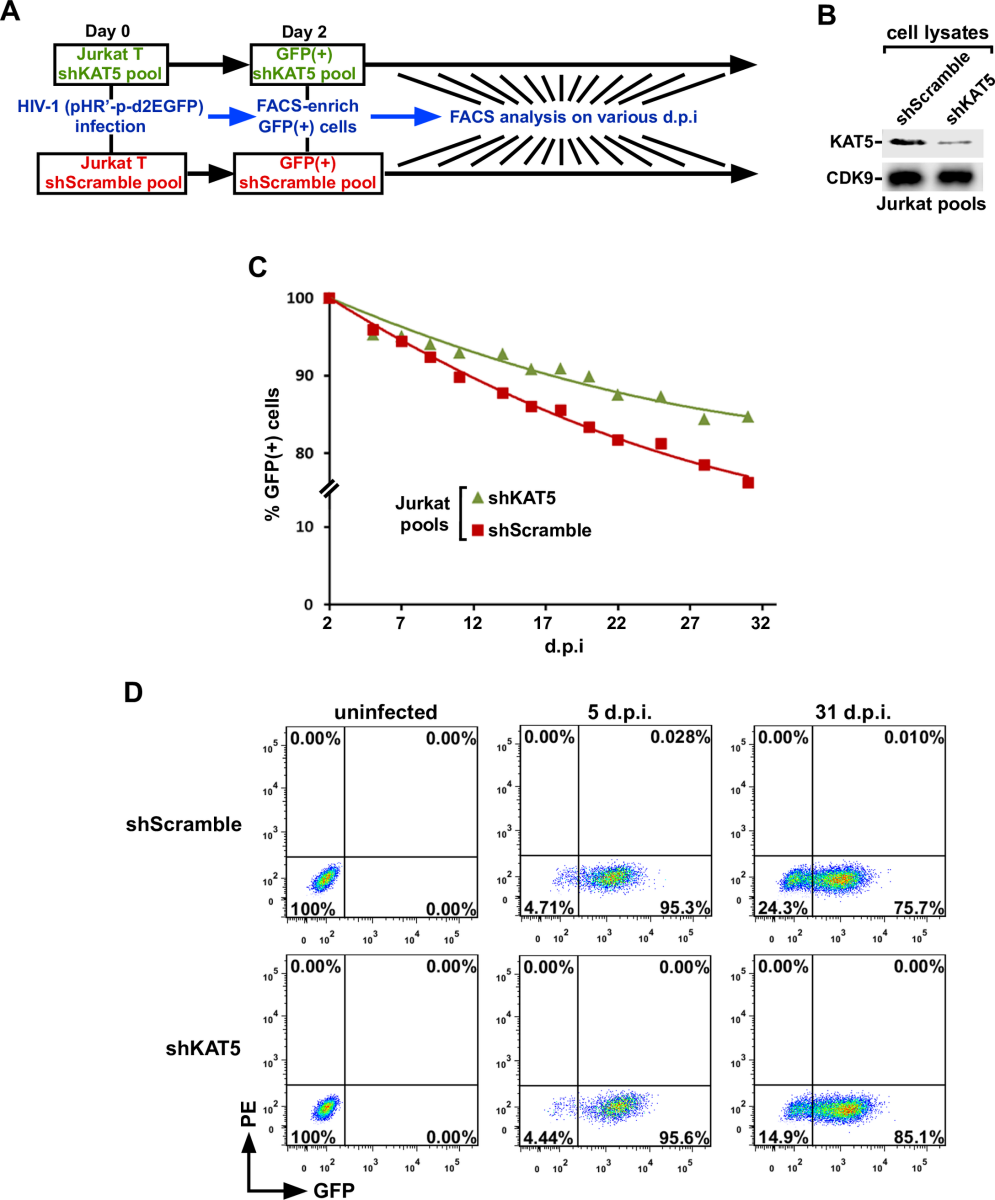
KAT5 depletion prevents HIV from efficiently establishing latency. **A.** A schematic diagram showing the procedure and timeline of experiments to progressively establish latency in Jurkat cell pools stably expressing either shKAT5 or shScramble. d.p.i.: Days post infection. **B.** Lysates of the cell pools expressing the indicated shRNA were examined by Western blotting for the proteins labeled on the left. **C.** The GFP(+) cells that were selected by FACS on 2 d.p.i from each cell pool were checked by flow cytometry for the percentages of GFP(+) cells on the indicated d.p.i. and the average of three measurements at each time point is shown. **D.** Representative immunofluorescence flow cytometry analysis of shScramble- and shKAT5-expressing cells that were either uninfected or infected with HIV and harvested at the indicated d.p.i.

The data presented thus far are consistent with the hypothesis that abundant AcH4 on the HIV provirus is used to locally recruit Brd4, which then competes with Tat for host P-TEFb. KAT5 enhances the levels of AcH4, and consequently it behaves as an inhibitor of the Tat-SEC formation on HIV LTR to suppress Tat-transactivation and enforce viral latency. Based on this notion, we hypothesized that silencing the expression of KAT5 would therefore make it more difficult for HIV to establish latency in the progressive latency establishment assay outlined in Fig 6A.

To test this hypothesis, we generated stable pools of Jurkat cells that express either a non-targeting scrambled shRNA (shScramble) or the KAT5-specific shRNA (shKAT5). The latter was shown to cause ~70% reduction in the cellular level of KAT5 (Fig 6B). When tested in the progressive latency establishment assay, the shKAT5 pool displayed a significantly delayed dynamics in reverting to the GFP(-) status, i.e. transcriptionally silent or latent state, when compared with the shScrambled pool (Fig 6C & 6D). This result confirms the prediction that KAT5 and its acetylation of H4 play a critical role in allowing HIV to efficiently establish latency.

### Inhibiting KAT5 potentiates proviral reactivation by JQ1 in a primary T cell-based latency model

**Fig 7:**
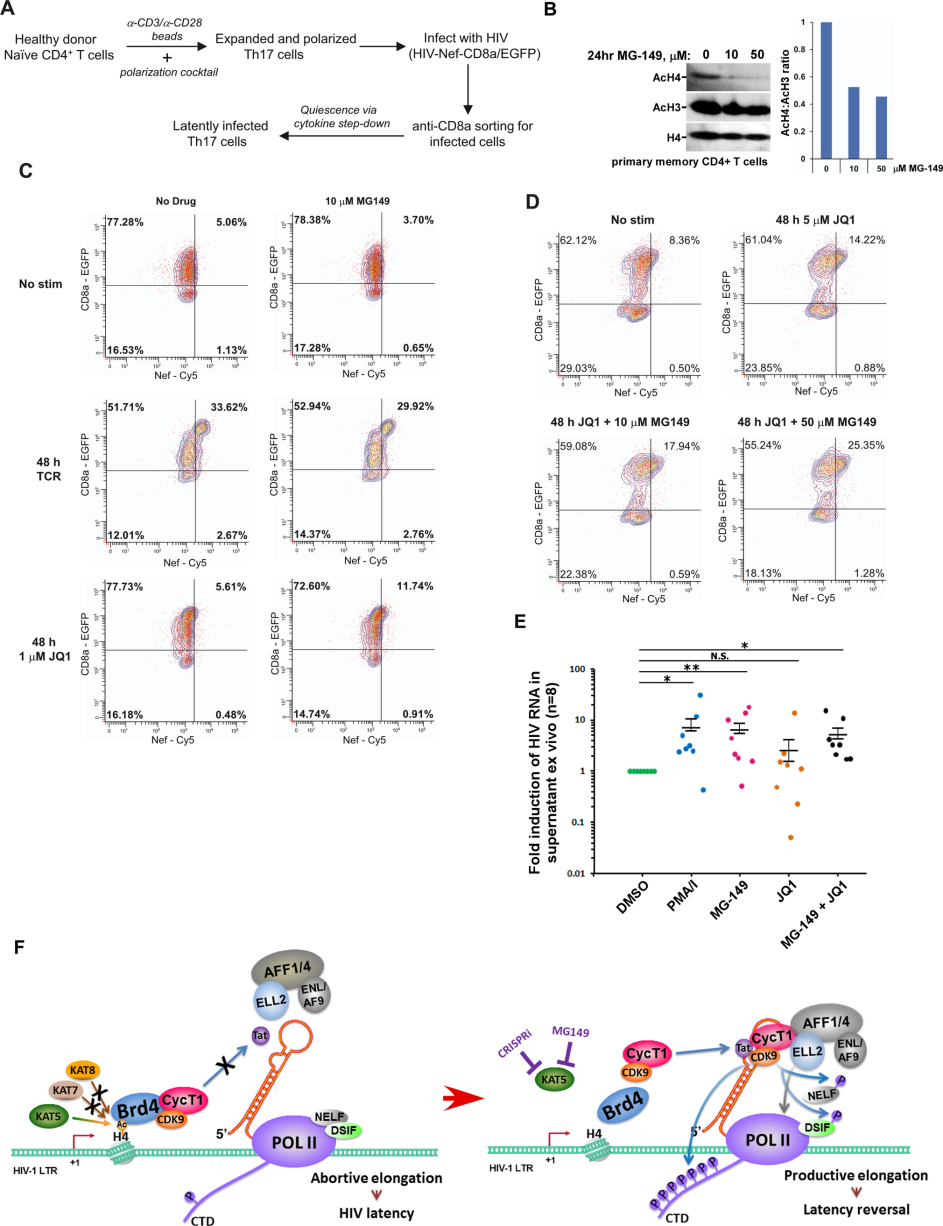
Inhibition of KAT5 in a primary cell latency model and ART-suppressed patient cells enhances HIV latency reversal and virion release. **A.** Scheme showing the procedure followed to generate latently infected resting Th17 cells. **B.** Memory CD4+ T cells from a healthy donor were pretreated with 1 μM SAHA overnight and then incubated with the indicated concentrations of MG-149 for 24 hr. Western blotting analyses were conducted to detect the levels of AcH4, AcH3, and total H4 in whole cell lysates. The AcH4:AcH3 ratio in each lane was measured by densitometry and shown on the right. **C.** Representative immunofluorescence flow cytometry analysis of Nef-Cy5 and CD8a-EGFP expression in latently infected primary Th17 cells that were either TCR-activated or treated with 1 μM JQ1 for 48 hr in the presence or absence of 10 μM MG-149. EGFP reporter used for positive selection remained high in the latently infected cells due to prolonged stability of the membrane-bound CD8a-EGFP fusion protein. **D.** Representative immunofluorescence flow cytometry analysis of Nef-Cy5 and CD8a-EGFP expression in latently infected primary Th17 cells that were either untreated or treated with 5 μM JQ1 with or without MG-149 for 48 hr. **E.** Primary cells from ART-suppressed HIV-1-infected individuals were treated with the indicated drug(s) for 24 hr. HIV-1 RNA in culture supernatant were quantified with the Roche COBAS AmpliPrep/TaqMan HIV-1 Qualitative Test system. The viral copy numbers were presented as fold induction relative to the DMSO control. Numbers in parentheses indicate number of individuals used for each treatment. N.S. not significant; *P < 0.05; **P < 0.01. Error bars represent standard error of the mean (SEM). **F.** Diagram depicting KAT5 but not KAT7 and KAT8 as a host factor for suppressing HIV proviral transcription and promoting viral latency. See text for more details.

In primary T-cells, P-TEFb levels are dramatically reduced when the cells enter quiescence, and this in turn, forces HIV into latency. To evaluate the role of KAT5 in maintaining HIV in a transcriptionally silent state in primary CD4+ T cells, we used our recently described Th17 cell latency model [36]. Briefly, polarized and expanding Th17 cells were infected with a VSVG-pseudotyped HIV-1 virus HIV-Nef-CD8a/eGFP and then forced to enter quiescence by culturing in a restrictive cytokine environment (Fig 7A). Proviral gene expression, as assessed by immunofluorescence staining for Nef, was low in the quiescent cells, but could be induced ~4 to 6-fold upon activation of the T-cell receptor (TCR) by antibodies to CD3 and CD28 (S5 Fig and Fig 7C). In each of these experiments, the EGFP signal remained high in Th17 cells containing latent HIV due to the prolonged stability of the membrane-bound CD8a-EGFP fusion protein.

As expected, MG-149 significantly and preferentially decreased the AcH4 level in primary CD4+ T cells (Fig 7B). In comparison, the levels of AcH3 and total H4 were only mildly reduced, probably due to an indirect effect of global inhibition of gene expression in the treated cells. When used alone, MG-149 (at 10 or 50 μM) and JQ1 (at 1 or 5 μM) produced no, or only very modest, stimulatory effects on proviral gene induction in our Th17 cell latency model (Fig 7C and 7D). However, exposure of the cells to 1 μM JQ1 plus 10 μM MG-149 for 48 hr increased the HIV-expressing cell population by more than 2-fold (Fig 7C). Similarly, while the treatment with 5 μM JQ1 alone slightly elevated HIV expression, the addition of 50 μM MG-149 further enhanced the stimulatory effect of 5 μM JQ1 by ~1.8-fold (Fig 7D). At 10 to 50 μM, MG-149 produced very little enhancing effect on HIV reactivation in cells that were treated with SAHA, the PKC agonist Ingenol [6,37], or TCR-activating signals (S5 Fig and Fig 7C). Thus, the enhancing effect of MG-149 appears to be restricted to JQ1 in this primary T cell model of latency. Of note, both MG-149 alone and in combination with other LRAs did not cause global T-cell activation as indicated by minimal changes in CD25 and CD69 immunostaining after the treatment (S6 Fig).

### Inhibition of KAT5 by MG-149 stimulates release of virions from primary T cells isolated from ART-treated patients

To further investigate the impact of KAT5 inhibition by MG-149 in a more clinically relevant setting, we performed an *ex vivo* experiment to test the effect of MG-149 alone or in combination with JQ1 to release virions from primary T cells that are isolated from ART-treated patients (Fig 7E and S1 Table). The data reveal a statistically significant positive effect displayed by MG-149 alone (6.5-fold increase on average of HIV RNA released into supernatant) as well as by the JQ1 plus MG-149 combination (5.3-fold increase on average). In comparison, JQ1 alone failed to produce a statistically significant effect.

It is interesting to note that a previous report shows that both the PKC-agonist and PMA plus ionomycin (PMA/I), but not JQ1 or HDACi alone, could efficiently increase virion release from latently infected primary cells [7]. Our *ex vivo* data confirm these observations about PMA/I and JQ1, and more importantly, show that MG-149 was almost as effective as PMA/I to single-handedly increase the virion release (Fig 7E). This effect of MG-149 could be important for maximally exposing the latently infected cells for immuno-recognition and clearance and thus implicate the potential utility of KAT5 inhibition as an effective latency reversal strategy.

## Discussion

In contrast to the well-characterized positive effects of histone H3 acetyltransferases p300/CBP and P/CAF on HIV transcription [4], the roles played by the major H4 acetyltransferases in this process are unknown. In this study, we report that KAT5, but not KAT7 and KAT8, is a host factor that promotes HIV latency establishment, and inhibits latency reversal, by acetylating H4 on the viral LTR. The unique pattern of histone acetylation found at the LTR permits recruitment of Brd4 to the HIV promoter where it competes with Tat for P-TEFb, blocks Tat-SEC formation, and ultimately inhibits Tat-transactivation (Fig 7F). When KAT5 is antagonized by either CRISPRi or MG-149, the loss of AcH4 on the LTR dissociates Brd4 and allows P-TEFb to join Tat in the Tat-SEC complex to enhance HIV transcriptional elongation and latency reversal (Fig 7F).

Importantly, the strong correlation between the shutdown of HIV transcription during latency establishment and elevated levels of AcH4 and Brd4 on the LTR was demonstrated not only with the extensively characterized Jurkat clone 2D10 cell model, but also in a pool of latently infected cells with diverse integration sites. Thus, the negative effects exerted by the KAT5-Ac-H4-Brd4 axis on HIV transcription are independent of the proviral integration site and the cellular chromatin context.

Although inhibitory to Tat-induced HIV transactivation, the KAT5-AcH4-Brd4 axis appears to play a positive role in stimulating the expression of cellular genes, especially those involved in primary response. The differential effects of KAT5 on transcription of the HIV versus host genes implicate this histone acetyltransferase as a promising candidate that can be selectively targeted to reactivate latent HIV without globally activating numerous host genes at the same time. The high selectivity toward HIV is likely to be an important attribute for LRAs used in the “Shock and Kill” strategy for eradicating latent HIV reservoirs [3].

In contrast to KAT5, KAT7 and KAT8 produced no inhibitory effects on HIV transcription and latency reversal (Fig 2D–2G). We ascribe this to KAT5’s broader substrate specificity toward four H4 lysines (K5, K8, K12, and K16) versus KAT8’s sole targeting of H4K16 and KAT7’s more restrictive target specificity [19,20,24]. It has been shown that the poly-acetylated K5, K8, K12 is essential for Brd4’s binding to H4, whereas the acetylation of K16 only minimally increases Brd4’s affinity to H4 peptides already acetylated at K5, K8, and K12 [8].

Interestingly, KAT5 was first identified as an HIV Tat-interacting protein of 60 kDa, hence originally named as Tip60 [38]. It was subsequently found to be inhibited by Tat [39]. Thus, in addition to the inhibition of KAT5 by MG-149, which functions as a LRA to kick-start the initial rounds of HIV transcription to produce viral proteins including Tat, the accumulated Tat protein may further inhibit KAT5 to decrease AcH4 and antagonize Brd4’s action, thus fueling another positive feedback loop besides the Tat-SEC axis to expedite the reversal of latency.

Proviral transcription of latent HIV is silenced by multiple mechanisms including heterochromatinization of the LTR [40]. In the present study, we found that the AcH3 level is very low on the LTR in 2D10 cells as well in a diverse population of latently infected Jurkat cells. In contrast, the AcH4 level on the LTR is relatively high and comparable to those on the cellular genes. The activity of AcH4 is therefore distinct from that of AcH3, which is an activating signal since it prevents H3K9/K27-methylations that recruit the chromatin-compacting proteins HP1 and Polycomb [23]. By contrast, AcH4 is compatible with heterochromatin [41] and can even induce heterochromatinization in certain cases [42]. Thus, our data point to a plausible scenario where latent HIV proviruses are low in AcH3 but high in AcH4, which helps keep the LTR heterochromatinized but still capable of retaining Brd4 to suppress Tat-transactivation. This also resolves an apparent paradox concerning how histone acetylation, which is generally considered pro-euchromatin and promote HIV transcription [18], can be exploited by Brd4 to further silence Tat-transactivation.

Finally, it is worth noting that prior to the current study, the KAT5-AcH4-Brd4 axis has already been reported as a restriction mechanism for papillomaviral and adenoviral gene transcription through retaining or interacting with negative transcription factors on the viral promoters [43,44]. In light of these findings, our current work adds HIV to the growing list of viral infections that employ KAT5 as a crucial regulator. Due to the presence of multiple additional restrictions imposed on proviral transcription in quiescent primary T cells, it is considerably more difficult to reverse HIV latency in these cells than in transformed cell lines [45]. Nevertheless, the observation that the KAT5 inhibitor MG-149 cooperates with JQ1 to promote HIV latency reversal not only in activated cell lines but also in quiescent primary T cells derived from ART-treated HIV patients underscores the clinical relevance of targeting the KAT5-AcH4-Brd4 axis. Future studies are necessary to explore this potential for therapeutic intervention and eventual eradication of HIV/AIDS.

## Materials and methods

### Ethics statement

The part of this study utilizing specimens from HIV-infected individuals has been approved by the Bioethics Review Committee of the Shenzhen Center for Disease Control and Prevention in China. All research participants gave written informed consent, and all subject data and specimens were coded to protect confidentiality.

### CRISPRi-induced downregulation of KAT5, KAT7 and KAT8

To downregulate KAT5, KAT7 and KAT8 in Jurkat 2D10 cells (previously generated by Karn lab based on human CD4+ T cells Jurkat line[21]), vectors pHR-TRE3G-Krab-dCas9-P2A-mCherry[25] and pLVX-advanced-TetOn (gift from IGI, UC Berkeley) were packaged separately using a 3^rd^ generation lentiviral packaging system and co-infected into Jurkat 2D10 cells. A clone (2D10-iI) expressing Krab-dCas9-HA-P2A-mCherry in response to doxycycline (Dox) treatment was picked by FACS, and verified by Western blot. DNA oligos containing sgKAT5#1 (sg1; 5’-GACTCAGTAGACCGCCAC-3’), sgKAT5#2 (sg2; 5’-GCCTCAGGCCGAGCCCTAGG-3’), sgKAT7#1 (sg1; 5'-GTGTATCAGTCCCAATCCTG-3'), sgKAT7#2 (sg2; 5'-GGGATCGTCCGCAGGATT-3'), and sgKAT8 (5’-GAGAGACGCGGCCCGGGGAT-3’) were synthesized and cloned separately into the pSico-BFP-puro vector[25], which was then packaged and transduced into the 2D10-iI cells. After 1 μg/ml puromycin selection for 4 days, the stable CRISPRi-KAT5-sg1, CRISPRi-KAT5-sg2, CRISPRi-KAT7-sg1, CRISPRi-KAT7-sg2, and CRISPRi-KAT8 cell pools were treated with 1 μg/ml Dox for 72 hours and checked by RT-qPCR and Western blot with KAT5 (Invitrogen), KAT7 (Bethyl) and KAT8 antibodies (Invitrogen) for CRISPRi efficiency.

### Reverse transcription and real-time PCR (RT-qPCR) assay

Total RNA from ~5×10^6^ cells were extracted by RNeasy kit (Qiagen) and reverse transcribed using the M-MLV Reverse Transcriptase (VWR) with random hexamers (Invitrogen). The cDNAs were subjected to qPCR using a DyNAmo HS SYBR Green qPCR kit (Fisher) on a CFX96 machine (Bio-Rad) with the following primers: qKAT5-F (5'-AACCAGGACAACGAAGATGAG-3'), qKAT5-R (5'-GTCACCCATTCATCCAGACG-3'); qKAT7-F (5'-AGCCCTTCCTGTTCTATGTTATG-3'), qKAT7-R (5'-CATAGCCCTGTCTCATGTACTG-3'), qKAT8-F (5'-GGGAAAGAGATCTACCGCAAG-3'), qKAT8-R (5'-TCCACGTCAAAGTACAGTGTC-3'); qActB-F (5'-AGAGCTACGAGCTGCCTGAC-3'), qActB-R (5'-AGCACTGTGTTGGCGTACAG-3'); qLTR-F (5’-GGGTCTCTCTGGTTAGACCAG-3’), qLTR-59R (5’-GGGTTCCCTAGTTAGCCAGAG-3’), qLTR-190R (5’-CTGCTAGAGATTTTCCACACTGAC-3’); qMYC-F (5'-TTCGGGTAGTGGAAAACCAG-3'), qMYC-R (5'-AGTAGAAATACGGCTGCACC-3'); qFOS-F (5'-TTGTGAAGACCATGACAGGAG-3'), qFOS-R (5'-CCATCTTATTCCTTTCCCTTCGG-3'); qJUNB-F (5'-AGCCCAAACTAACCTCACG-3'), qJUNB-R (5'-GGGCATCGTCATAGAAGGTC-3'). All reactions were carried out in triplicates. The PCR signals were normalized to those of ActB and displayed.

### Cell line-based latency reversal assay

The CRISPRi-KAT5-sg1/sg2, CRISPRi-KAT8, or parental Jurkat 2D10 cells were first treated with the various LRAs or MG-149 (APExBIO) at the indicated concentrations for 20 hr, and then subjected to flow cytometry on a BD Bioscience LSR Fortessa X20 cytometer for GFP fluorescence. The data were analyzed using Flowjo software. Each drug treatment was done in 200 μl RMPI medium (Invitrogen) with 10% FBS (Gemini 900108 Lot A96C). To induce CRISPRi-mediated downregulation of KAT5 or KAT8, the CRISPRi-KAT5-sg1/sg2, CRISPRi-KAT8 cells were pre-treated for 48 hr with 1 μg/ml Dox before the LRA treatments. For control groups, 0.1% DMSO was used.

### Generation of latently infected quiescent Th17 primary T cells

Naïve CD4+ T cells isolated from a previously frozen healthy donor leukapheresis pack (previously generated by Karn lab[36]) by negative selection using the EasySep Naïve CD4 T-cell isolation kit (19155RF; Stem Cell) were simultaneously treated with T-cell receptor activator magnetic beads and a Th17 polarization cytokine cocktail for 6 days as previously described[36]. On Day 4, IL-2 was added to the cells at 60 IU/ml. Following Th17 polarization and during T-cell expansion, cells were either infected or not with a VSVG-pseudotyped pHR’-Nef+-CD8a/GFP viral construct by spinoculation. HIV-infected cells were later positively selected for by anti-CD8a magnetic separation. To generate quiescent T cells, infected cell populations were cultured in medium containing reduced concentrations of IL-2 (15 IU/ml) and IL-23 (12.5 μg/ml) for at least 2 weeks. Proviral latency was monitored by assessing for the expression of Nef and gp120 Env by immunofluorescence flow cytometry before and after T-cell receptor reactivation for 18 hr. Achievement of resting T cells was monitored by immunostaining for Ki67, cyclin D3, and pSer175 CDK9 under the same conditions.

### Flow cytometry analysis of proviral reactivation in latently infected primary T cells

Primary resting Th17 cells, latently infected with HIV as described above, were pretreated or not for 30 min with MG-149 at varying concentrations (10 or 50 μM) prior to 24 or 48 hr challenge with either of the following stimuli: T-cell receptor activator anti-CD3/anti-CD28 antibody cocktail, JQ1 (at 1 μM or 5 μM), SAHA (500 nM), or ingenol (20 nM). Cells were washed once with 1X PBS and fixed in 4% formaldehyde for 15 min at room temperature prior to permeabilization with 1X BD Perm/Wash buffer (BD Biosciences). After blocking with a non-specific IgG for 15 min, cells were immunofluorescently stained for HIV Nef. Thereafter, these cells were washed three times with 1X permeabilization buffer and subjected to flow cytometry analysis using the LSR Fortessa instrument (BD Biosciences) equipped with the appropriate laser and filters.

### Western blotting analysis of MG-149’s effect on AcH4 levels in primary T cells

Approximately 2.4 × 10^7^ memory CD4+ T cells were isolated by negative selection from a healthy donor leukapheresis pack containing 1.5 × 10^8^ PBMCs. Following the isolation, cells were allowed to recover overnight in 10 ml complete RPMI media supplemented with 60 IU/ml IL-2 and 1 mM SAHA and then divided equally into a 24-well plate and treated with varying concentrations of MG-149 for 24 hr. Whole cell extracts were mixed with 50 ml of a 2X SDS-PAGE sample buffer prior to microtip sonication to mechanically shear viscous DNA. Thereafter, the samples were boiled at 95°C for 10 min and subjected to SDS-PAGE and immunoblotting analysis for N-terminally acetylated histone H4, total histone H4, and N-terminally acetylated histone H3. Densitometry analysis was performed using Quantity One software (Bio-Rad).

### Measurement of HIV-1 mRNA in culture supernatants of primary cells from ART-treated patients

HIV-1–infected individuals enrolled in the study were based on the criteria of good response to suppressive ART and undetectable plasma HIV-1 RNA levels. The PBMCs from the EDTA^+^ blood were isolated by Ficoll centrifugation. Approximately 10 × 10^6^ PBMCs were cultured in 2 ml RPMI1640 medium containing 10% FBS. The cells were then treated with 0.2% DMSO, 50 ng PMA + 1 μM Ionomycin (PMA/I) as a positive control, 50 μM MG-149, 2 μM JQ1, or 50 μM MG-149 + 2 μM JQ1 for 24 hr. After spinning down the cells, supernatants (1 ml each) were collected and subjected to HIV-1 RNA quantification with the Roche COBAS AmpliPrep/TaqMan HIV-1 Qualitative Test system. Each sample was tested twice, and the average viral copy numbers were normalized to the DMSO group and displayed in a scatter plot.

### Stable shRNA knockdown (KD) of KAT5 in Jurkat-based cells

The Jurkat 1G5[30] and 1G5+Tat cells[46] (both kind gifts from Melanie Ott lab, Gladstone Institutes, San Francisco) were infected with the pLKO.1-puro-based lentiviral vector containing shScramble 5’-CCTAAGGTTAAGTCGCCCTCG-3’, or shKAT5 5'-CCTTGACCATAAGACACTGTA-3' sequences. Two days after infection, the cells were selected by 1 μg/ml puromycin for 5 days until the stable pools were obtained. The KD efficiencies of the pools were examined by Western blot.

### Luciferase reporter assay

Two days before the assay, HEK 293T cells (from American Type Culture Collection) at ~60% confluency in 6-well plates were transfected using Polyethylenimine in triplicates by 0.1 μg HIV-1 LTR-luciferase construct combined with 1.85 μg pcDNA3-FLAG-KAT5[47] and/or 0.05 μg pRK5-Tat-HA. The total amount of DNA in each transfection was brought to 2 μg by empty pcDNA3 vector when necessary. For luciferase assays in WT Jurkat 1G5 or 1G5+Tat cells, 1 ml cells at the concentration of 0.5 million/ml were aliquoted into 12-well plates in triplicates and treated with the indicated concentrations of MG-149 for 18 hr. For luciferase assays in 1G5-/+Tat stable shRNA cell pools, the cells were counted on a haemocytometer and about 1.7 million cells from each pool were aliquoted in triplicates. The cells were harvested and lysed in 200 μl 1 × Reporter Lysis Buffer (Promega), and centrifuged at maximum speed on a benchtop centrifuge for 30 seconds. The supernatants were subjected to luciferase activity measurement by using the Luciferase Assay System (Promega) on a Lumat LB 9501 luminometer. For each group, 10 μl cell lysate was aliquoted before centrifugation from each repeat and pooled for Western blots to check the indicated protein levels.

### Chromatin immunoprecipitation (ChIP) assay

The assay was based on Nelson *et al*.[48] with some modifications. Briefly, 10 million cells were fixed by 1% formaldehyde for 10 min at room temperature, and then quenched for 5 min by adding glycine to 125 mM final concentration. After washing with PBS and lysed in the IP buffer[48], the chromatin was sheared using the Covaris S220 System with 200 Watt peak energy for 30 cycles (30 sec ON, 20 sec OFF) to an average size of 0.5–1 kb DNA fragments. The sheared chromatin was centrifuged at 20,800 × g for 10 min at 4°C. Each ChIP reaction was carried out with 300 μl supernatant and 1 μg each of the following antibodies: anti-Ac-H3 (Millipore, 06–599), anti-Ac-H4 (Millipore, 06–598), anti-H3 (Invitrogen, PA5-31954), anti-H4 (Invitrogen, 720166), anti-FLAG (Sigma, F1804), anti-Brd4 [10], normal rabbit total IgG (Santa Cruz, sc-2027), and normal mouse total IgG (Santa Cruz, sc-2025). After overnight incubation with rotation at 4°C, the reactions were centrifuged at 20,800 × g for 10 min, and 90% of each supernatant was combined with 20 μl Salmon sperm DNA (Invitrogen)-blocked Protein A Agarose (Invitrogen). After 45 min rotation, the beads were washed 6 times with 1 ml IP buffer and DNA fragments were extracted by boiling in Chelex (Bio-Rad). qPCRs were carried out with the following primers:

HIV-nuc1-F (5'-CTGGGAGCTCTCTGGCTAACTA-3'),

HIV-nuc1-R (5'-TTACCAGAGTCACACAACAGACG-3');

HIV-env-F (5'-TGAGGGACAATTGGAGAAGTGA-3'),

HIV-env-R (5'-TCTGCACCACTCTTCTCTTTGC-3');

MYC-C-F (5’-GCGCGCCCATTAATACCCTTCTTT-3’),

MYC-C-R: (5’-ATAAATCATCGCAGGCGGAACAGC-3’);

IκBα 5’end-F (5'-AAGAAGGAGCGGCTACTGGAC-3'),

IκBα 5’end -R (5'-TCCTTGACCATCTGCTCGTACT-3');

HERVK-LTR-F (5'-GGGCAGCAATACTGCTTTGT-3'),

HERVK-LTR-R (5'-CAATAGTGGGGAGAGGGTCA-3');

intergenic-F (5'-CTCCCAAATTGCTGGGATTA-3'),

intergenic-R (5'-ATTCCAGGCACCACAAAAAG-3').

For ChIP in NH1 cells (previously generated by Zhou lab based on human HeLa cell line [49]) containing an integrated HIV-1 LTR-luciferase reporter construct, the cells seeded in 6-well plates were first transfected in duplicate by either 2 μg empty vector or pCMV2-based vectors containing N-terminally FLAG-tagged Brd4 long isoform (Brd4L, isoform A, amino acids [aa] 1–1,362), or Brd4 short isoform (Brd4S, isoform C, aa 1–722). Both Brd4 vectors were kind gifts from Melanie Ott lab (Gladstone Institutes, San Francisco, [50]). 31 hours after transfection, DMSO or MG-149 were added to the medium to the final concentrations of 0.1% and 30 μM respectively. After 18 hr of drug treatment, about 10^6^ cells/well were subjected to ChIP as described above. The ChIP was conducted using anti-FLAG beads (Sigma, A2220), and the qPCRs were carried out with the following primers: LTRS-1 (5’-GTTAGACCAGATCTGAGCCT-3’), and LTRS-2 (5’-GTGGGTTCCCTAGTTAGCCA-3’).

Signals obtained by qPCR were normalized to those of input DNA, and the averages from triplicated qPCR reactions were shown with the error bars representing standard deviations. Two-tailed Student's *t*-tests were conducted and the different significance levels were marked by 1 to 3 asterisks.

### HIV-1 latency establishment assay

The HIV-1 infection and progressive latency establishment assay were based on Pearson *et al*.[21] with some modifications. Briefly, HIV-1 virions were produced by transfecting HEK 293T cells with the wild-type pHR’-p-d2EGFP plasmid[21] using a 3^rd^ generation lentiviral packaging system and then used to spin-infect wild-type Jurkat (from American Type Culture Collection) or Jurkat pools expressing the indicted shRNAs in the presence of 6 μg/ml polybrene (Santa Cruz). Two days post infection (d.p.i.), GFP(+) cells (of the HIV-infected Jurkat cells described above) were enriched by flow cytometry and allowed to propagate in culture. The percentages of GFP(+) cells in this population (called the total pool in Fig 1) were monitored continuously until the indicated d.p.i. On 29 d.p.i., a population of GFP(-) cells were enriched by flow cytometry from the original GFP(+) cells selected on 2 d.p.i. and maintained as the latent pool in Fig 1. On 43 d.p.i., the latent pool was subjected to latency reversal assays as described above with the indicated concentrations of LRAs, and both the total and latent pools were subjected to Western blots and ChIP assays for the indicated proteins.

## Supporting information

S1 Fig CRISPRi inhibition of KAT5 expression by using an alternative sgRNA (sg2) that targets a different KAT5 promoter sequence produces the same result as in CRISPRi-KAT5-sg1 cells. **A.** The Jurkat 2D10-based inducible CRISPRi-KAT5-sg2 cells were treated with (+) or without (-) Dox and analyzed by RT-qPCR for the KAT5 mRNA levels, which were normalized to those of ActB. **B., C. & D.** CRISPRi-KAT5-sg2 cells were treated with or without Dox (1 μl/ml) and the various LRAs at the indicated concentrations, and then subjected to FACS analysis to determine the percentage of GFP(+) cells in each cell population.
(TIF)

S2 Fig CRISPRi inhibition of KAT7 expression by using an alternative sgRNA (sg2) that targets a different KAT7 promoter sequence produces the same result as in CRISPRi-KAT7-sg1 cells. **A.** The Jurkat 2D10-based inducible CRISPRi-KAT7-sg2 cells were treated with (+) or without (-) Dox and analyzed by RT-qPCR for the KAT5 mRNA levels, which were normalized to those of ActB. **B., C., & D.** CRISPRi-KAT7-sg2 cells were treated with or without Dox (1 μl/ml) and the various LRAs at the indicated concentrations, and then subjected to FACS analysis to determine the percentage of GFP(+) cells in each cell population.
(TIF)

S3 Fig Antagonizing KAT5 synergizes with JQ1 to promote HIV transcription at largely the elongation stage. Top: a schematic diagram showing the elements of HIV-1 5' LTR and the positions of transcription start site (TSS) and the primer pairs used in RT-qPCR reactions to quantify the short 59-nucloetide (nt) and long 190-nt HIV-1 transcripts. Bottom: CRISPRi-KAT5-sg1 and the parental 2D10 cells were treated with the indicated drugs. Total RNAs extracted from these cells were subjected to RT-qPCR quantifications to determine the short and long HIV-1 transcripts using the indicated specific primers. The qPCR signals were normalized to those of ActB. Each column represents the average of three independent RT-qPCR reactions, with the error bars indicating mean +/- SD.
(TIF)

S4 Fig On a per-molecule basis, more Brd4 binds to HIV LTR than does Brd4S and the Brd4-LTR binding is also more sensitive to MG-149-induced AcH4 reduction. NH1 cells containing an integrated HIV-1 LTR were transfected with either an empty vector or vectors expressing the indicated FLAG-tagged Brd4 isoforms, treated by either 0.1% DMSO or 30 μM MG-149 for 18 hr, and subjected to ChIP-qPCR analysis using the anti-FLAG beads to determine the levels of the Brd4 isoforms bound to HIV LTR. The ChIP-qPCR signals were normalized to those of input DNA. The error bars represent mean +/- SD from three independent qPCR reactions. An aliquot of each cell sample was also examined by Western blotting for the proteins labeled on the left.
(TIF)

S5 Fig MG-149 fails to potentiate the effect of SAHA, Ingenol or T-cell receptor activation on proviral reactivation in a primary T cell model of latency. **A.** Latently infected Th17 cells (No stim) were placed in media containing 60 IU/ml IL-2 and then challenged with MG-149 for 24 hr in the presence or absence of SAHA (500 nM), ingenol (20 nM), or α-CD3 antibody (500 ng/ml). Proviral HIV expression was determined by flow cytometry measurements of the percentage of cells that were positive for both Nef and EGFP. Graphed data are from two independent experiments. **B.** Latently infected Th17 cells were stimulated or not with an antibody cocktail of α-CD3/α-CD28 for 24 or 48 hr in the absence or presence of the indicated concentrations of MG-149. Proviral HIV expression was determined by flow cytometry measurements of the percentages of cells positive for both Nef and EGFP. Graphed data for the 24 hr treatment are from four independent experiments and the 48 hr treatment from two experiments.
(TIF)

S6 Fig MG-149 does not induce global T cell activation. Primary resting CD4^+^ T cells were treated for 24 hr with the indicated drugs or their combinations. The levels of T cell activation were accessed by immunostaining of CD25 and CD69, which was then analyzed by flow cytometry.
(TIF)

S1 Table Characteristics of HIV-1–infected study participants. (DOC)
