# Supplementary figures and images for: The KAT5-Acetyl-Histone4-Brd4 axis silences HIV-1 transcription and promotes viral latency
Source: PLoS Pathog. 2018 Apr 23;14(4):e1007012. doi: 10.1371/journal.ppat.1007012 (PMC5933813; doi:10.1371/journal.ppat.1007012)

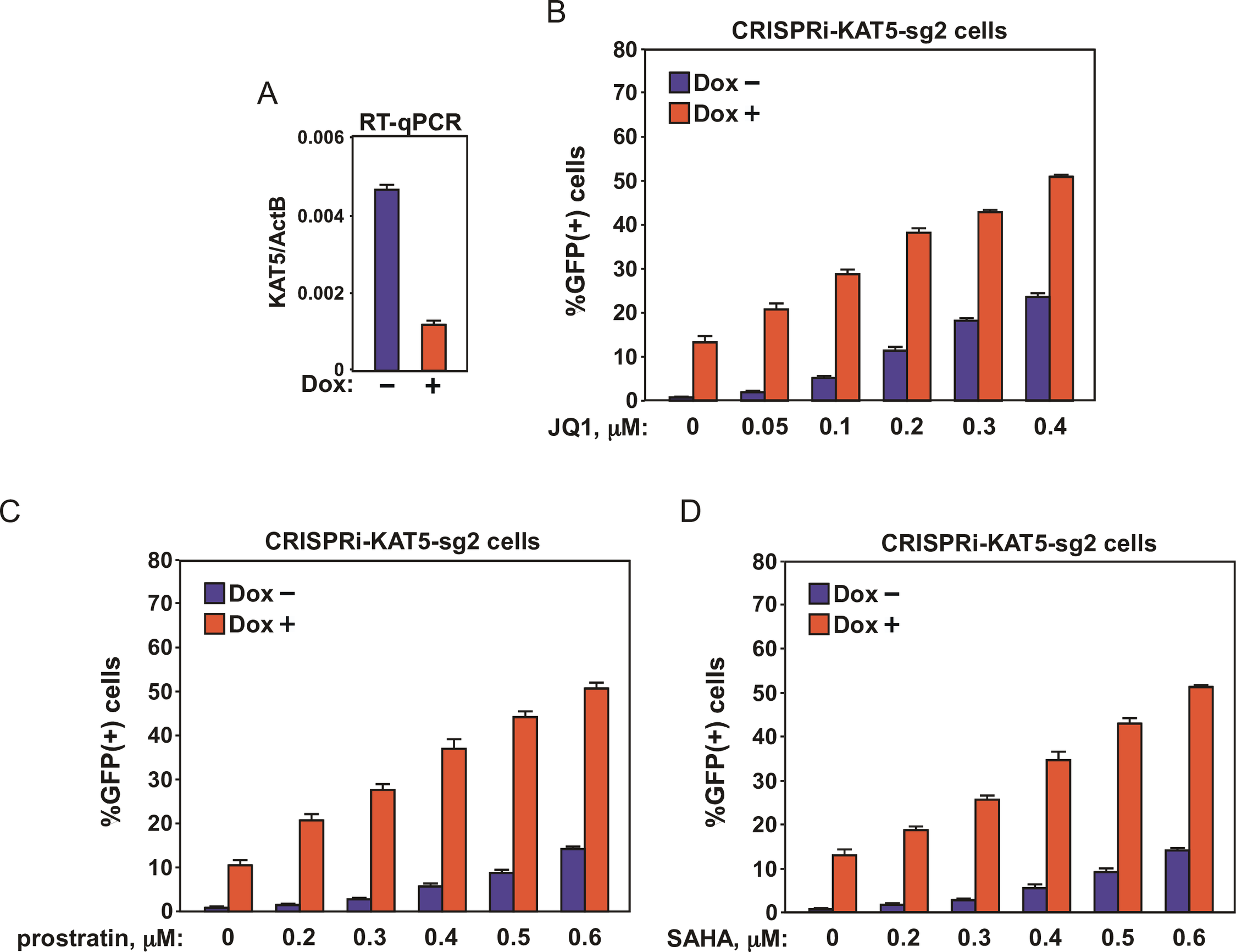

Supplement: S1 Fig — A. The Jurkat 2D10-based inducible CRISPRi-KAT5-sg2 cells were treated with (+) or without (-) Dox and analyzed by RT-qPCR for the KAT5 mRNA levels, which were normalized to those of ActB. B., C. & D. CRISPRi-KAT5-sg2 cells were treated with or without Dox (1 μl/ml) and the various LRAs at the indicated concentrations, and then subjected to FACS analysis to determine the percentage of GFP(+) cells in each cell population. (TIF) [file ppat.1007012.s001.tif]

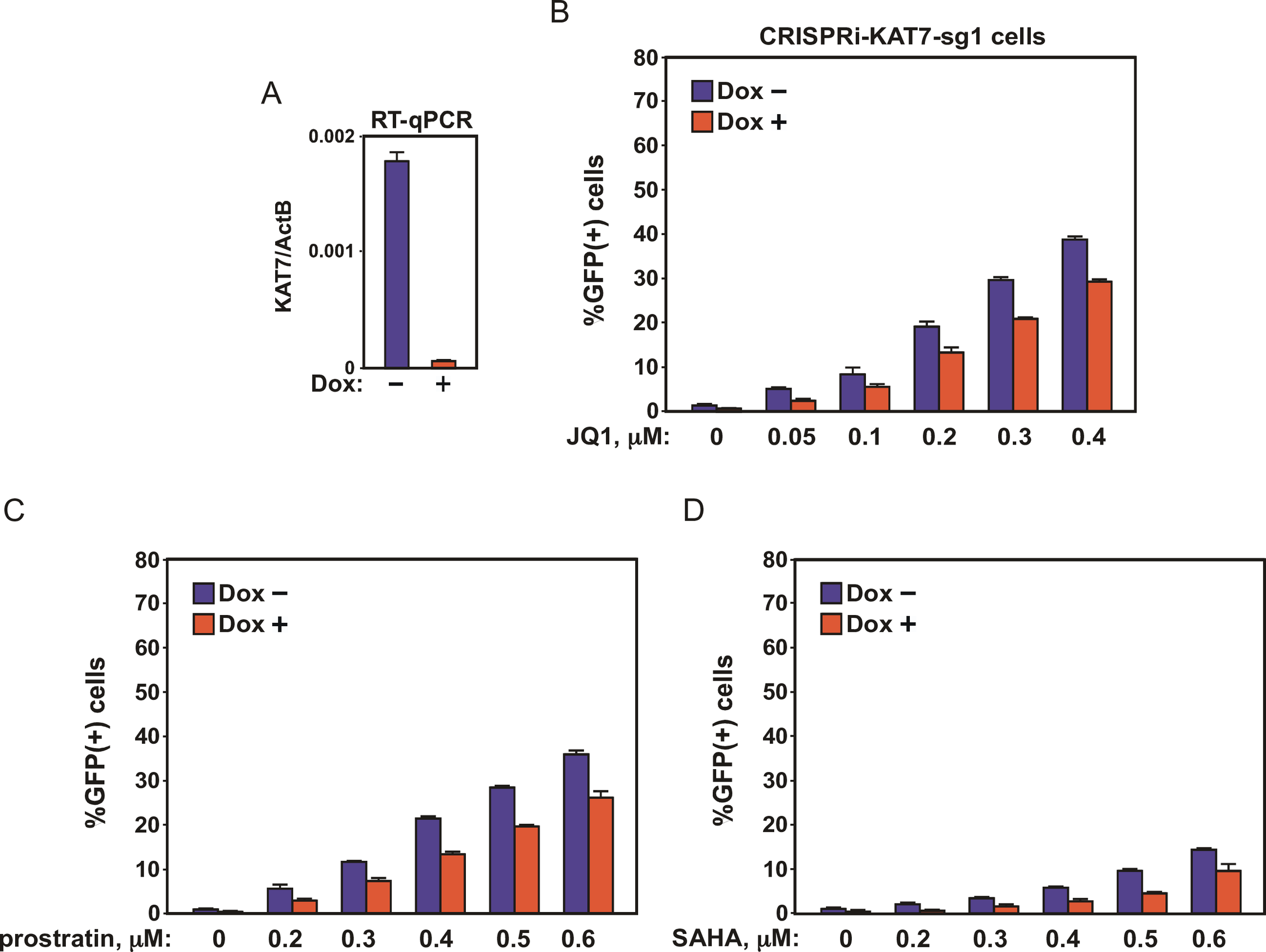

Supplement: S2 Fig — A. The Jurkat 2D10-based inducible CRISPRi-KAT7-sg2 cells were treated with (+) or without (-) Dox and analyzed by RT-qPCR for the KAT5 mRNA levels, which were normalized to those of ActB. B., C., & D. CRISPRi-KAT7-sg2 cells were treated with or without Dox (1 μl/ml) and the various LRAs at the indicated concentrations, and then subjected to FACS analysis to determine the percentage of GFP(+) cells in each cell population. (TIF) [file ppat.1007012.s002.tif]

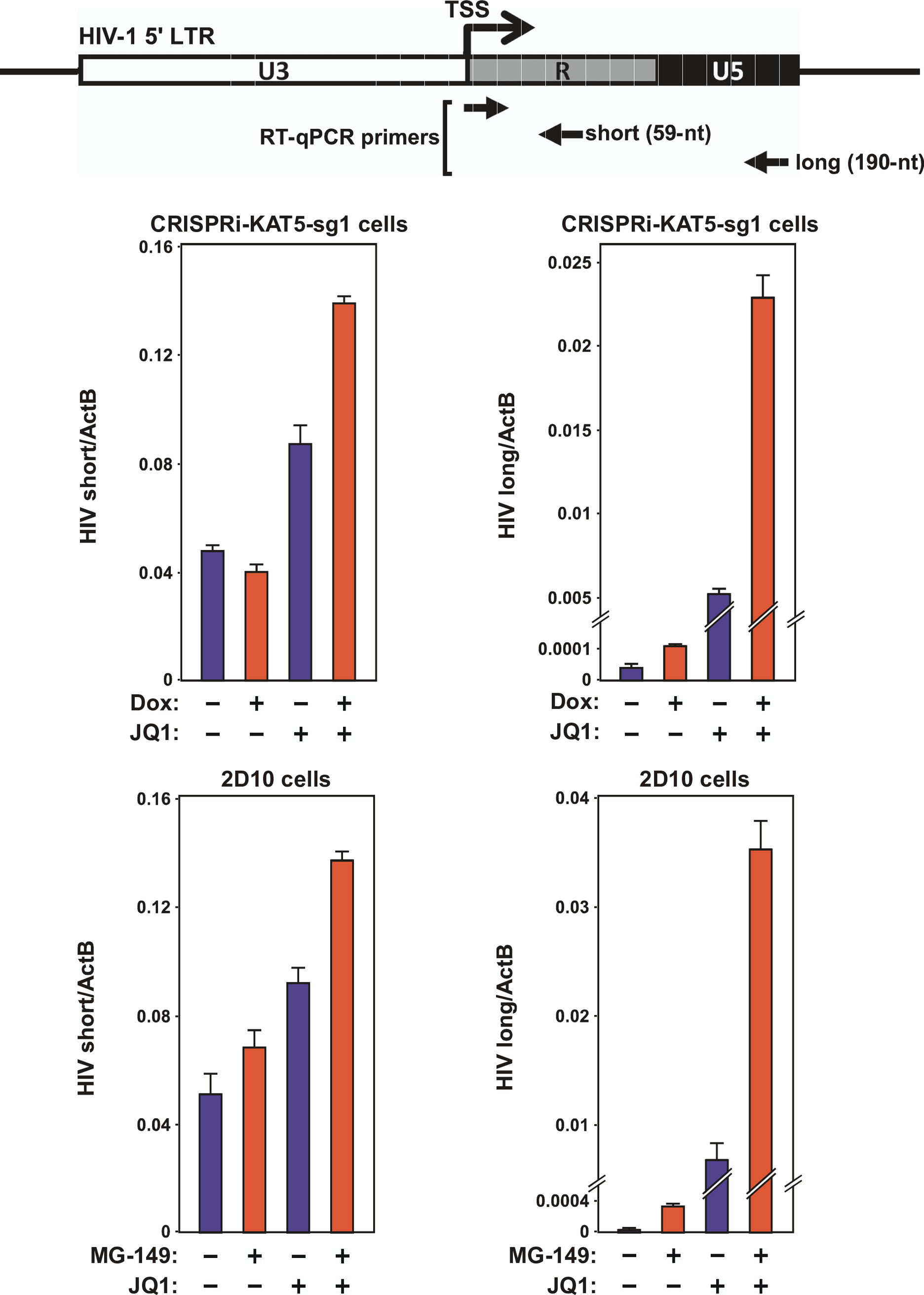

Supplement: S3 Fig — Top: a schematic diagram showing the elements of HIV-1 5' LTR and the positions of transcription start site (TSS) and the primer pairs used in RT-qPCR reactions to quantify the short 59-nucloetide (nt) and long 190-nt HIV-1 transcripts. Bottom: CRISPRi-KAT5-sg1 and the parental 2D10 cells were treated with the indicated drugs. Total RNAs extracted from these cells were subjected to RT-qPCR quantifications to determine the short and long HIV-1 transcripts using the indicated specific primers. The qPCR signals were normalized to those of ActB. Each column represents the average of three independent RT-qPCR reactions, with the error bars indicating mean +/- SD. (TIF) [file ppat.1007012.s003.tif]

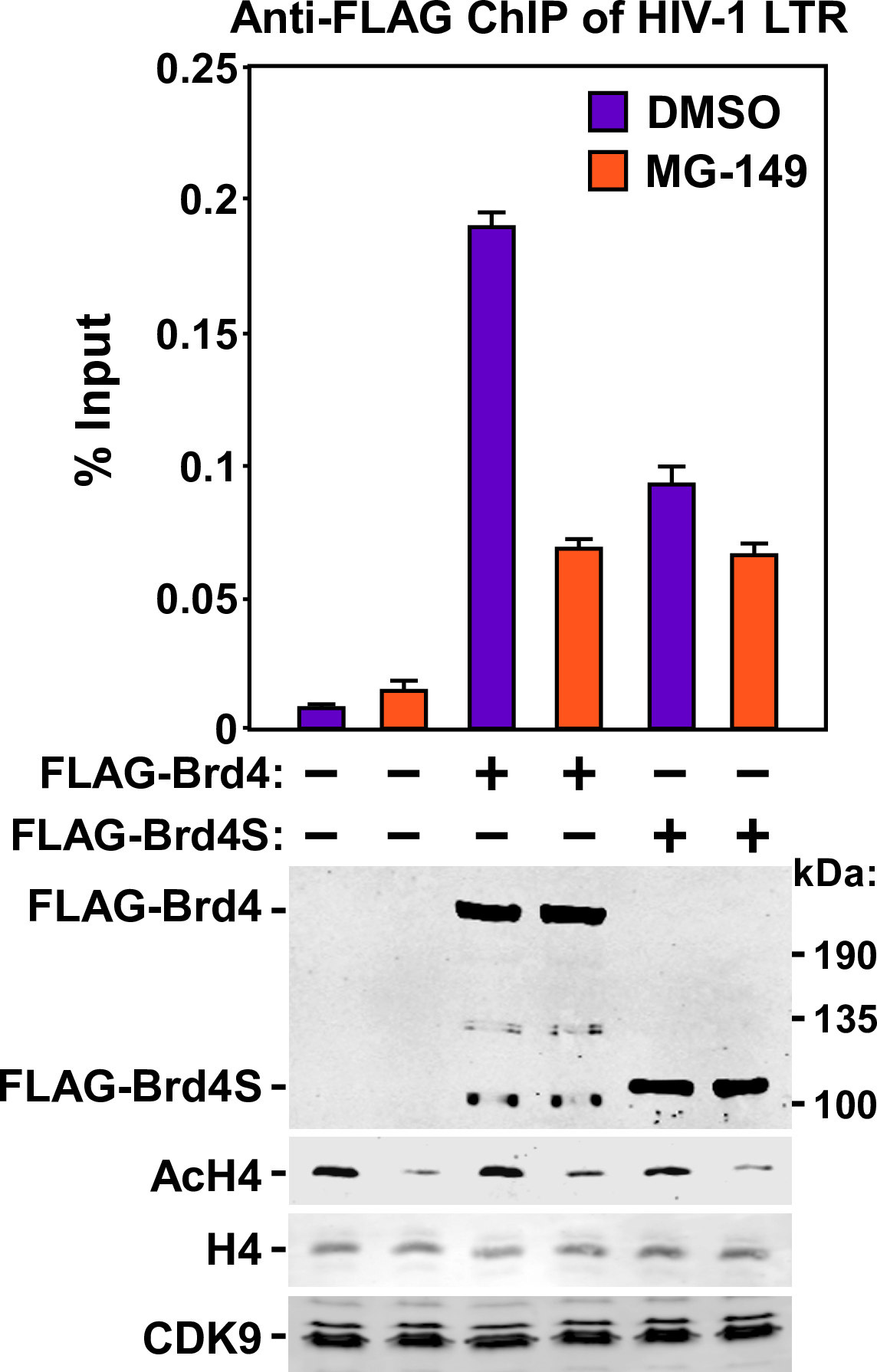

Supplement: S4 Fig — NH1 cells containing an integrated HIV-1 LTR were transfected with either an empty vector or vectors expressing the indicated FLAG-tagged Brd4 isoforms, treated by either 0.1% DMSO or 30 μM MG-149 for 18 hr, and subjected to ChIP-qPCR analysis using the anti-FLAG beads to determine the levels of the Brd4 isoforms bound to HIV LTR. The ChIP-qPCR signals were normalized to those of input DNA. The error bars represent mean +/- SD from three independent qPCR reactions. An aliquot of each cell sample was also examined by Western blotting for the proteins labeled on the left. (TIF) [file ppat.1007012.s004.tif]

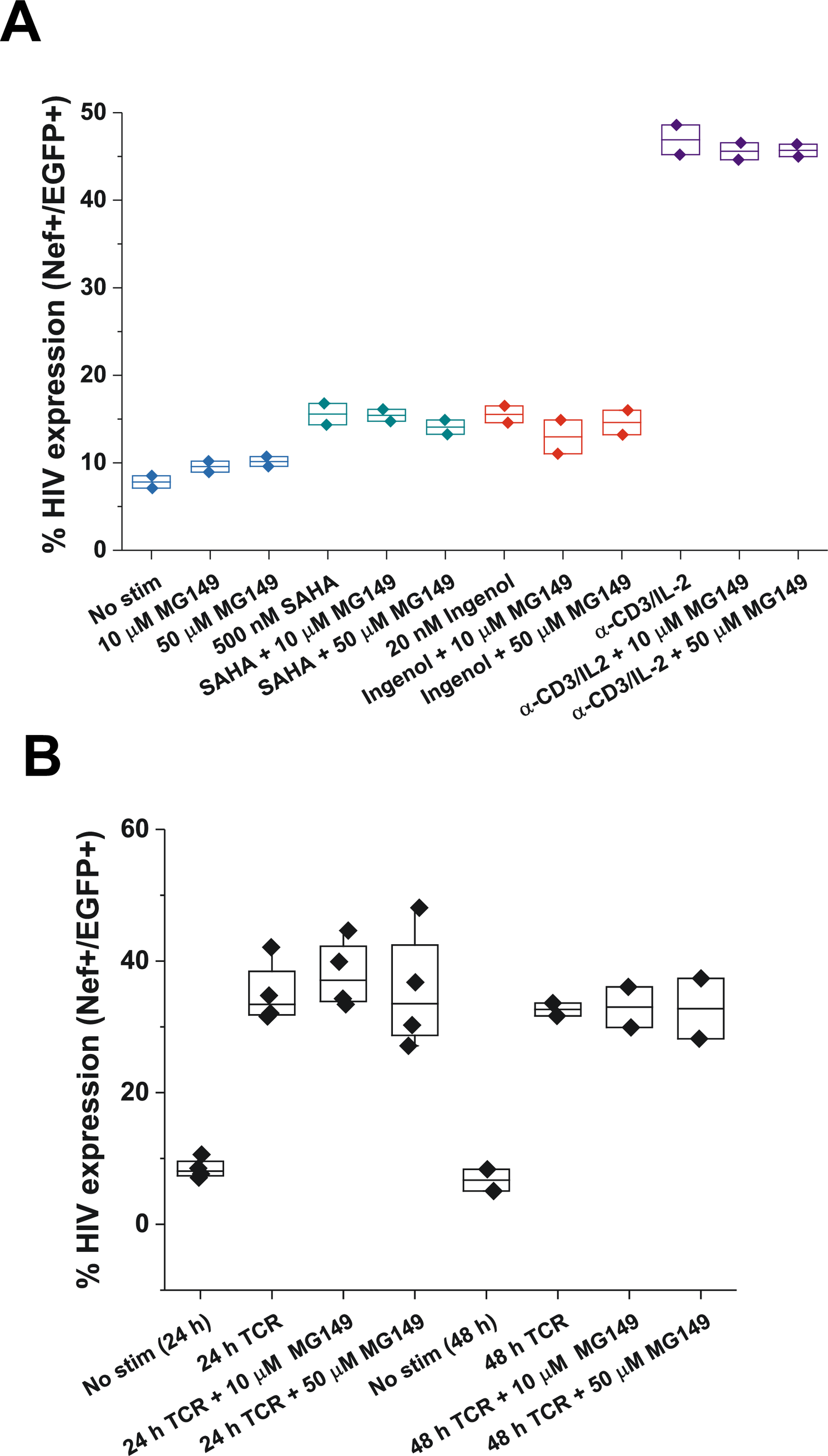

Supplement: S5 Fig — A. Latently infected Th17 cells (No stim) were placed in media containing 60 IU/ml IL-2 and then challenged with MG-149 for 24 hr in the presence or absence of SAHA (500 nM), ingenol (20 nM), or α-CD3 antibody (500 ng/ml). Proviral HIV expression was determined by flow cytometry measurements of the percentage of cells that were positive for both Nef and EGFP. Graphed data are from two independent experiments. B. Latently infected Th17 cells were stimulated or not with an antibody cocktail of α-CD3/α-CD28 for 24 or 48 hr in the absence or presence of the indicated concentrations of MG-149. Proviral HIV expression was determined by flow cytometry measurements of the percentages of cells positive for both Nef and EGFP. Graphed data for the 24 hr treatment are from four independent experiments and the 48 hr treatment from two experiments. (TIF) [file ppat.1007012.s005.tif]

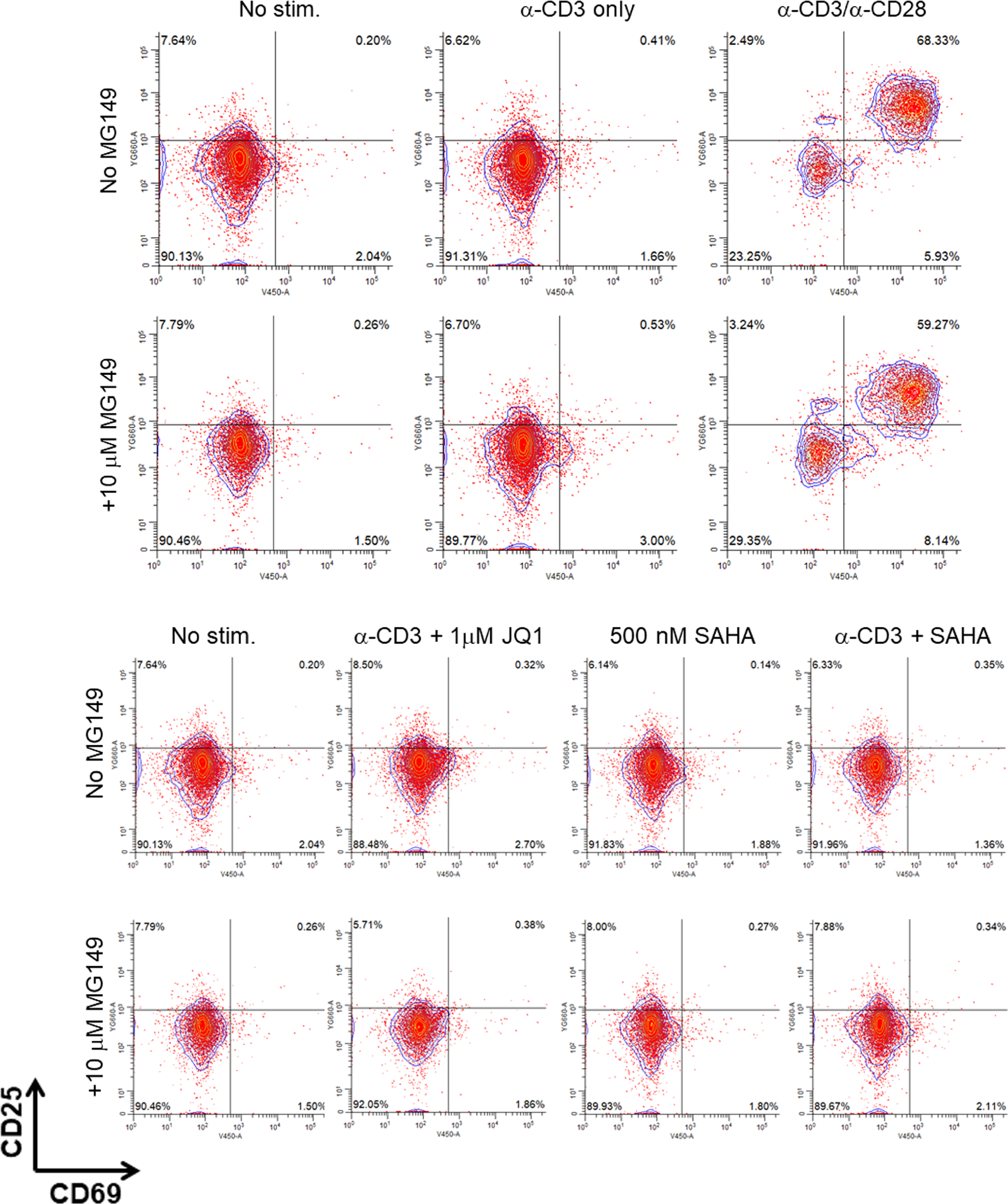

Supplement: S6 Fig — Primary resting CD4+ T cells were treated for 24 hr with the indicated drugs or their combinations. The levels of T cell activation were accessed by immunostaining of CD25 and CD69, which was then analyzed by flow cytometry. (TIF) [file ppat.1007012.s006.tif]
