# Supplementary material for: The KAT5-Acetyl-Histone4-Brd4 axis silences HIV-1 transcription and promotes viral latency
Source: PLoS Pathog. 2018 Apr 23;14(4):e1007012. doi: 10.1371/journal.ppat.1007012 (PMC5933813; doi:10.1371/journal.ppat.1007012)
Supplement: S1 Table — (DOC) [file ppat.1007012.s007.doc]

**S1 Table. Characteristics of HIV-1–infected study participants.**

| ID | Age | Sex | Race | Duration of infection (months) | ART regimen | Time on ART (months) | Time on suppressive ART (months) | CD4 count (cells/mm^3^) | Peak reported viral load (copies ml^–1^) |
| --- | --- | --- | --- | --- | --- | --- | --- | --- | --- |
| 1 | 29 | M | A | 24 | EFV/3TC/TDF | 24 | 21 | 543 | 3.55 × 10^4^ |
| 2 | 37 | M | A | 24 | EFV/3TC/TDF | 24 | 15 | 179 | 9.19 × 10^5^ |
| 3 | 35 | M | A | 38 | EFV/3TC/TDF | 13 | 9 | 697 | 3.52 × 10^5^ |
| 4 | 39 | F | A | 90 | EFV/3TC/TDF | 65 | 33 | 819 | Unknown |
| 5 | 35 | M | A | 7 | EFV/3TC/TDF | 6 | 3 | 536 | 1.42 × 10^5^ |
| 6 | 34 | M | A | 7 | EFV/3TC/TDF | 6 | 3 | 517 | 2.56 × 10^4^ |
| 7 | 47 | F | A | 38 | EFV/3TC/TDF | 36 | 26 | 440 | 2.42 × 10^4^ |
| 8 | 52 | M | A | 119 | EFV/3TC/AZT | 118 | 81 | 620 | unknown |

M, male; F, female; A, Asian; 3TC, lamivudine; TDF, tenofovir disoproxil fumarate; EFV, efavirenz; AZT, azidothymidine.
